# Physiographic Environment Classification: a Controlling Factor Classification of Landscape Susceptibility to Waterborne Contaminant Loss

**DOI:** 10.1007/s00267-024-01950-0

**Published:** 2024-03-05

**Authors:** Clinton W. F. Rissmann, Lisa K. Pearson, Ton H. Snelder

**Affiliations:** 1 Land and Water Science, Invercargill, New Zealand; 2 Waterways Centre for Freshwater Management, University of Canterbury, and Lincoln University, Christchurch, New Zealand; 3 LWP Ltd, Christchurch, New Zealand

**Keywords:** Controlling factor landscape classification, Hydrochemical maturity, Susceptibility to contaminant loss, Water quality, Environmental management

## Abstract

Spatial variation in the landscape factors climate, geomorphology, and lithology cause significant differences in water quality issues even when land use pressures are similar. The Physiographic Environment Classification (PEC) classifies landscapes based on their susceptibility to the loss of water quality contaminants. The classification is informed by a conceptual model of the landscape factors that control the hydrochemical maturity of water discharged to streams. In New Zealand, a case study using climatic, topographic, and geological data classified the country into six, 36, and 320 classes at Levels 1 (Climate), 1–2 (Climate + Geomorphology), and 1–3 (Climate + Geomorphology + Lithology), respectively. Variance partitioning analysis applied to New Zealand’s national surface water monitoring network (*n* = 810 stations) assessed the contributions of PEC classes and land use on the spatial variation of water quality contaminants. Compared to land use, PEC explained 0.6× the variation in Nitrate Nitrite Nitrogen (NNN), 1.0× in Total Kjeldahl Nitrogen (TKN), 1.8× in Dissolved Reactive Phosphorus (DRP), 2.3× in Particulate Phosphorus (PP), 2.6× in *E. coli*, and 4.3× in Turbidity (TURB). Land use explained more variation in riverine NNN, while landscape factors explained more variation in DRP, PP, *E. coli*, and TURB. Overall, PEC accounted for 2.1× more variation in riverine contaminant concentrations than land use. The differences in contaminant concentrations between PEC classes (*p* < 0.05), after adjusting for land use, were consistent with the conceptual model of hydrochemical maturation. PEC elucidates underlying causes of contaminant loss susceptibility and can inform targeted land management across multiple scales.

## Introduction

Contaminant losses from agricultural land use are a major driver of poor water quality, degraded ecosystems, and risks to human health (Boyd 2019; Larned et al. 2020; Snelder et al. 2023). Even where land use intensities are similar, the type of water quality degradation (e.g., dissolved vs. particulate) and its severity is spatially variable (Becker et al. 2014; O’Sullivan et al. 2023). Much of this variability is due to spatial variation in environmental factors, including climate, geomorphology, and lithology, which interact across multiple scales to influence the type and severity of waterborne contaminant loss (Becker et al. 2014; Lintern et al. 2018; O’Sullivan et al. 2023).

Landscape classifications are used in many environmental management activities as spatial frameworks for interpreting data, extrapolating information from specific sites to larger areas, developing objectives, standards/regulations, and designing monitoring strategies. Landscape classifications that are based on controlling factors are assumed to describe the cause of patterns in characteristics of interest, such as ecosystems (e.g., Bailey 1995), hydrological regimes (e.g., Snelder et al. 2005), or water quality (e.g., Krantz and Powars 2002). This type of landscape classification codifies the understanding of processes that determine the characteristics of distinct patches on the Earth’s surface, including the susceptibility to certain types of impacts, which assists in defining management actions that are appropriate and specific (Christensen et al. 1996). Despite the potential benefits for environmental management, the development of a holistic and systematic classification based on the factors controlling susceptibility to the generation, retention, loss, and attenuation of waterborne contaminants has been limited.

In this article, we describe the Physiographic Environment Classification (PEC), which is a novel approach to classifying and mapping “landscape units” (i.e., patches on the Earth’s surface) that have distinct susceptibilities to waterborne contaminant loss. The classification of contaminant susceptibility is informed by a conceptual model of the landscape factors that determine the hydrochemical maturity of the waters produced by landscape units. PEC is based on the proposition that while land use is the primary driver of degraded water quality, spatial variation in the susceptibility of landscape units to the loss of different water quality contaminants is determined by several processes, including climatic, hydrological, biogeochemical, and mechanical. PEC is based on the assumption that environmental factors, including climate, geomorphology, and lithology, are the dominant controls on these processes.

The following text elaborates on the PEC’s conceptual model, clarifying and expanding on the aforementioned concepts and those of earlier studies (Rissmann et al. 2018, 2019). A case study implementation of PEC at a national scale is presented for the diverse landscape of New Zealand, utilizing commonly available spatial data, including climatic, topographic, and geological maps. We aim to show, using New Zealand’s national surface water monitoring network, that PEC can discriminate variation in multiple water quality indicators that are associated with agricultural land use, i.e., including nitrate-nitrite-nitrogen (NNN), organic and ammoniacal nitrogen (TKN), dissolved reactive forms of phosphorus (DRP), particulate phosphorus (PP), turbidity (TURB), and *E. coli*.

## Method

### PEC Conceptual Model

**Fig. 1:**
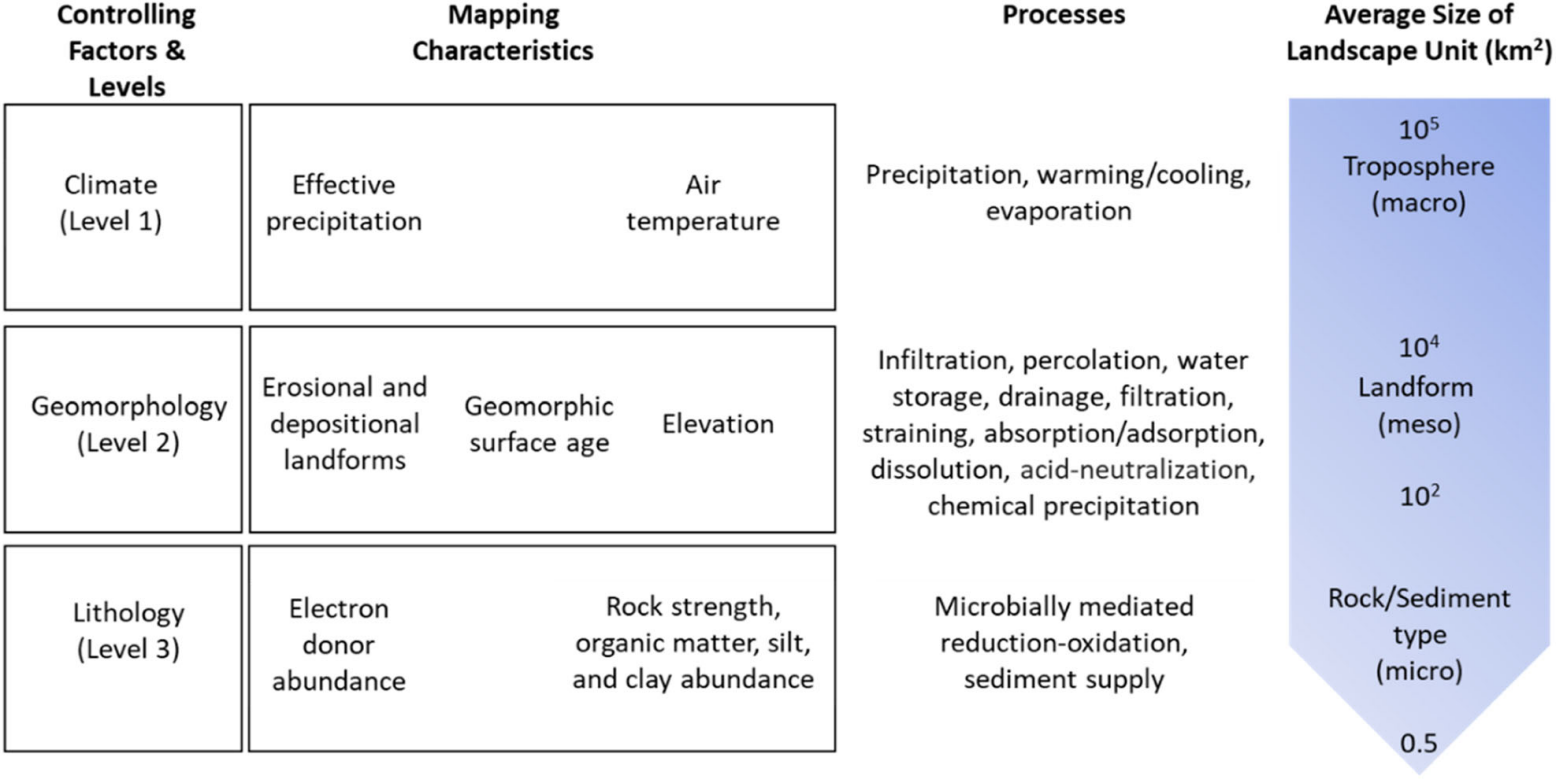
The conceptual model underpinning PEC is based on processes that determine the hydrochemical maturity of water produced by a landscape unit at three hierarchically organized system levels. Each level is defined by a “controlling factor,” which is assumed to be the dominant cause of patterns at the associated spatial scale

The guiding principles (*sensu* Zonneveld 1994) for controlling factor classifications start with a conceptual model of the causes of patterns in the characteristics of interest (Fig. 1). The rationale is that a classification is more useful if classes are defined according to a model of the causes of patterns in characteristics rather than by direct observation of the characteristics (e.g., water quality measurements) themselves (Bailey 1995). The conceptual model is a pragmatic simplification of reality that allows us to “approach the truth by a series of approximations” (Bailey 1995).

A further guiding principle is that patterns and processes are hierarchically organized (Fig. 1). The hierarchy refers first to an assumed “dominance in spatial scale” (Klijn 1994). This principle expresses the idea that landscape units are homogeneous, but homogeneity is not absolute and depends on the scale of observation (O”Neill et al. 1989). The hierarchy also refers to “dominance in process,” which expresses the idea that the magnitudes of processes at one system level constrain the behavior of the system level below (see Section 2.1.3).

The concept of landscape factors controlling processes and causing patterns in characteristics of interest at specific scales or system levels is consistent with our understanding of the influence of the landscape on the hydrochemical maturation of freshwater (e.g., Drever 1997; Robinson and Kapo 2003; Clark and Fritz 2013). It is understood that waters that are exposed to a similar set of environmental factors, including climate, geomorphology, and lithology, undergo similar processes and subsequently share a similar range of physical, chemical, isotopic, microbial, biomolecular characteristics, and degree of hydrochemical maturity (Güler and Thyne 2004). PEC uses the controlling factor approach to landscape classification of Bailey (1998) and Klijn and de Haes (1994) to classify landscapes according to the variation in the processes that determine the hydrochemical maturity of the waters they produce.

We define hydrochemical maturity as the extent to which precipitation intercepted by the land surface and eventually discharged from a landscape unit has undergone modification due to its interaction with regolith materials. The interaction between precipitation and the regolith, commonly referred to as “water-rock interaction,” also accounts for biologically mediated transformations and reactions with organic matter (Hem 1970; Drever 1997; Clark and Fritz 2013). The magnitude of water-rock interaction is typically indicated by the abundance of the major rock-forming elements (mainly as cations) and associated reaction products (mainly as anions). All other things being equal, i.e., assuming the same rock or sediment type and degree of weathering, low levels of mineralization, characterized by low concentrations of the major rock-forming elements and associated reaction products, are indicative of low levels of interaction between precipitation and the regolith. Conversely, high levels of mineralization, which are characterized by higher concentrations of the major rock-forming elements and associated reaction products, are indicative of high levels of interaction between precipitation and the regolith. Therefore, precipitation that has extensively interacted with the regolith is described as more mineralized or “hydrochemically mature.” In contrast, precipitation that has experienced minimal interaction with the regolith is characterized as weakly mineralized or “hydrochemically immature”.

Four approximations underpin our conceptual model of the causes of hydrochemical maturity. First, patterns in hydrochemical maturity are caused by spatial variation in processes, including precipitation, warming/cooling, evaporation, infiltration, percolation, water storage, drainage, filtration, straining, detachment and mobilization (including detachment and entrainment of surficial debris and regolith materials by flowing water), absorption/adsorption, dissolution, chemical precipitation, acid-neutralization, and microbially mediated reduction-oxidation (redox). Second, these processes are controlled by three independent “controlling factors”: climate, geomorphology, and lithology. Third, the same processes that control water’s hydrochemical maturity determine a landscape unit’s susceptibility to the generation, transformation, retention, loss, or attenuation of a wide range of contaminants. Fourth, the magnitude of processes responsible for the hydrochemical maturation of water can be ordered to discriminate variation in the maturity (least to most mature) of the waters produced and the susceptibility (low to high) of landscape units to contaminant loss.

Our conceptual model comprises three system levels, which are defined by the factors that are assumed to be the dominant cause of patterns in hydrochemical maturity at each level: Climate (Level 1), Geomorphology (Level 2), and Lithology (Level 3) (Fig. 1). As applied here, geomorphology encompasses topography and the texture, thickness, and intensity of weathering of the regolith. Variation in the controlling factors at each level is differentiated by categories. The categories are used to classify and map “landscape units” with characteristic sizes that we refer to as macro-, meso-, and microscale. PEC classes at each system level are defined by concatenating the controlling factor categories assigned to that unit for that and all preceding levels.

Hierarchical organization means that PEC delineates patterns in hydrochemical maturity and susceptibility to contaminant loss with increasing resolution at successive classification levels, and the internal variability of large-scale patches is resolved by lower levels (Klijn and de Haes 1994). When mapped, PEC classes recur across the geographic domain as non-contiguous landscape units. At a catchment level, the drainage network receives inputs from multiple landscape units belonging to differing PEC classes so that the hydrochemical maturity and contaminant characteristics of waters at any point of the network can be understood as the integration of contributions from all upstream landscape units.

The conceptual model assumes that the processes that control the hydrochemical maturation of water occur within the regolith. We define the regolith as the unconsolidated material overlying unweathered bedrock, including: i. the uppermost soil or “pedolith”; ii. alluvium and other transported “cover materials” (e.g., wind deposited, volcanic air fall, glacial till/outwash, mass wasting deposits); iii. “saprolith” as oxidized and chemically reduced rock and sediment, and; iv. “saprock” as fractured bedrock with weathering restricted to fracture margins. Our definition of regolith includes the unconfined groundwater system, where groundwater is defined as water that exists beneath the pedolith within a zone of saturated cover materials (e.g., alluvium, volcaniclastics), weathered bedrock (saprolith) or weakly weathered bedrock (saprock). The model only pertains to portions of the shallow groundwater table connected to the surface water network. Confined aquifers or deeper parts of an unconfined aquifer that are poorly hydrogeologically connected to the surface water network or do not discharge as spring-fed streams are not part of the classification.

#### Agricultural contaminants

Agricultural contaminants are deposited onto the land surface, generated at the land surface in response to mechanical disturbance, or generated within the topsoil and shallow subsoil in response to abiotic and biotic processes. The main agricultural contaminants include: pathogens (e.g., *E. coli*); organic N and P both as particulate organic and dissolved organic forms; inorganic N as ammoniacal N, nitrite (NO_2_^-^), and nitrate (NO_3_^-^); inorganic P as orthophosphate (PO_4_^3-^), and; nutrient and pathogen enriched silts, clays, and organic matter.

Once in the regolith, agricultural contaminants maybe transformed into other forms (e.g., urea to nitrate) or they are attenuated (e.g., plant uptake and export, denitrification, die-off of pathogens). The transformation and attenuation characteristics of agricultural contaminants vary with their physiochemical character, the environmental conditions that are encountered (e.g., temperature, moisture content; Schimel et al. 2007), and the time spent in contact with the regolith (Fetter et al. 1999; McMahon and Chapelle 2008; Tan 2009). All other things being equal, contact time and environmental conditions are controlled by the combination of landscape factors within the area being farmed. Generally, contaminants that spend greater lengths of time in contact with the regolith, under favorable environmental conditions, are more likely to be transformed or attenuated, than those that are restricted to short contact times or unfavorable environmental conditions (Fetter et al. 1999; Austin et al. 2004; Schimel et al. 2007). Consequently, landscape factors discriminate contaminant forms and associated susceptibilities to loss.

We define primary contaminants as particulate contaminants derived from manures, vegetable matter, pathogens, and the silt and clay of agriculturally enriched pedolith. If contact times and environmental conditions are favorable, primary contaminants are converted to secondary, and, ultimately, tertiary contaminants.

The silts and clays of agriculturally enriched regolith are characterized by elevated phosphorus, nitrogen, and pathogens bound at particle surfaces or sequestered internally relative to undeveloped regolith (Brady and Weil 2008; Tan 2009). Decomposing vegetable matter has a strong, pH-dependent ability to absorb and adsorb both pathogens and nutrients. Organic matter originating from pastoral or crop species, which may make up a significant proportion of the soil organic matter (SOM) pool, is characterized by greater concentrations of nitrogen and phosphorus within the molecular structure of the vegetative materials (i.e., lower C:N and C:P ratios), relative to endemic flora and SOM (Chen and Chen 2021). Agriculturally enriched silt, clay, or organic matter, lost from the landscape and that accumulate as bed sediments, may smother benthic habitat, drive sediment anoxia, and act as slow-release fertilizers, emitting adsorbed and absorbed secondary and tertiary nutrients into the overlying water column (Stutter et al. 2018).

Secondary contaminants are derived from primary contaminants. Secondary contaminants include short-lived compounds that are rapidly transformed into tertiary contaminants if contact times and the environmental conditions encountered within the regolith are favorable. For instance, ammonium (NH_4_^+^) and nitrite (NO_2_^-^) are secondary (or intermediate) contaminants generated during nitrification. Similarly, organic forms of N (including deposited urine and synthetic urea) and P (e.g., inositol hexaphosphate or phytic acid) are also secondary contaminants. However, the secondary forms of P are typically less bioavailable, requiring greater contact times and specific environmental conditions for transformation to tertiary orthophosphate relative to the secondary forms of N (Moldan and Cerny 1994; Tan 2009).

Tertiary contaminants represent the endpoint of the biogeochemical transformation of primary or secondary contaminants into their simplest and most bioavailable forms. Nitrate is a tertiary contaminant and is the ultimate end product of the nitrification of primary (plant organic matter) or secondary (ammonium and urea) contaminants. Orthophosphate is also a tertiary contaminant, it is the end product of the mineralization of primary (plant organic matter, manures, or mineral phosphate) or secondary (e.g., phytic acid) contaminants.

The transformation of primary to secondary, and secondary to tertiary contaminants may take weeks, months, or years under favorable environmental conditions (Oberson et al. 2005; Richardson et al. 2009; Tan 2009). Likewise, the attenuation of contaminants via filtering, straining, absorption/adsorption, chemical precipitation, or microbially mediated redox processes occur over similar time frames (Moldan and Cerny 1994; Fetter et al. 1999; McMahon and Chapelle 2008; Tan 2009).

In agricultural systems, nitrate and orthophosphate are also deposited or injected at the regolith surface as synthetic fertilizers. Synthetic fertilizers consist of highly soluble salts of nitrate and orthophosphate (e.g., sodium nitrate (NaNO_3_), triple superphosphate (Ca(H_2_PO_4_)_2_), diammonium phosphate ((NH_4_)_2_HPO_4_), or soluble and rapidly metabolized molecular precursors such as urea (CO(NH_2_)_2_). Unlike nitrogen (N) and phosphorus (P) derived from plant materials, manure, or weathering of the regolith, synthetic fertilizers are more soluble and reactive, requiring much shorter contact times for transformation under favorable environmental conditions into labile secondary (such as ammoniacal-N, urea) and tertiary contaminant forms. However, where landscape factors favor short contact times or environmental conditions are unfavorable, synthetic fertilizers may be lost before being absorbed/adsorbed by regolith surfaces or assimilated by growing plants. In their solid state, we view synthetic fertilizers as primary contaminants, which may be lost in response to overland flow or rapidly solubilized and leached if heavy rainfall occurs soon after application.

#### Water maturity and susceptibility to contaminant loss

Hydrochemical maturity refers to the stage of geochemical evolution of a body of water as determined by its composition, particularly the composition of regolith-derived solutes (i.e., major and trace ions). The concept is commonly applied in groundwater sciences, to describe changes in water hydrochemistry in both space and time (Drever 1997; Güler and Thyne 2004; Clark and Fritz 2013). Many of the same processes that control the hydrochemical maturation of water also control the generation, transformation, and attenuation of water quality contaminants and the suitability of water for ecological, agricultural, industrial, and domestic uses (Hem 1970; Winter et al. 1998; Fetter et al. 1999; Boyd 2019).

Put simply, the hydrochemical maturation of the water produced by a landscape unit depends on the time water spends in contact with the regolith (Maher 2010; Sterte et al. 2021; Burt et al. 2022). And contact time is a function of the climate, infiltration capacities, depth of percolation, hydraulic gradients, the flowpath length, and the hydraulic conductivity encountered along the flowpath (Maher 2010; Sterte et al. 2021; Burt et al. 2022). Consequently, as with the transformation of contaminants (“Agricultural contaminants”), the hydrochemical maturity of waters produced by a landscape unit is fundamentally controlled by landscape factors, which include climate, topography, and the texture and thickness of regolith (Moldan and Cerny 1994; Maher 2010; Tetzlaff et al. 2009).

**Fig. 2:**
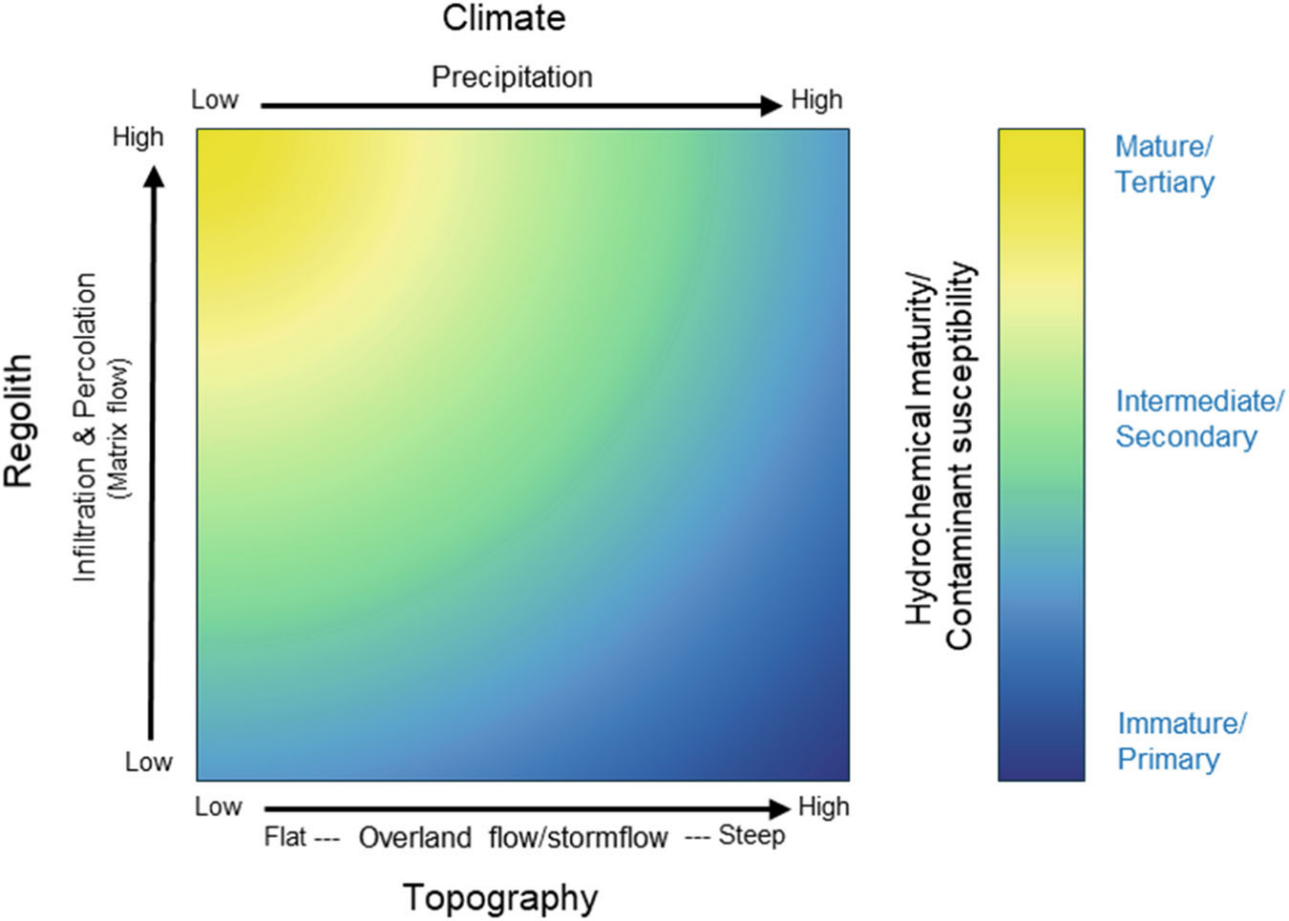
Schematic of variation in hydrochemical maturity and susceptibility to contaminant loss along controlling factor gradients. The hydrochemical maturity of water produced by the landscape is controlled by Climate and Geomorphology (as defined by topography and the texture, thickness, and intensity of weathering of the regolith). Susceptibility to primary, secondary, and tertiary contaminant loss is most elevated where hydrochemically immature, intermediate, and mature waters are produced, respectively. Landscape units exhibit varying degrees of temporal variation in the hydrochemical maturity of the waters they produce, and therefore, susceptibility to the loss of different contaminant forms

Climatic processes such as precipitation, heating/cooling, and evaporation control the hydrochemical maturation of water and the susceptibility of landscape units to contaminant loss. Landscape units in areas of high precipitation volume are less likely to produce hydrochemically mature waters due to shorter contact times and higher rates of dilution, whereas the converse is true of landscape units within areas of low precipitation volume (Drever 1997; Clark and Fritz 2013). All other things being equal, shorter contact times within high precipitation volume areas are expected to favor the loss of primary contaminants, whereas the production and loss of tertiary contaminants is expected where precipitation volume is lower (Fig. 2). Solar radiation-driven evaporation concentrates solutes and contaminants, amplifying reaction rates that control the hydrochemical maturation of water (Clark and Fritz 2013), and the transformation of contaminants from primary through to tertiary forms (Tan 2009; Boyd 2019). Abiotic and biotic reaction rates are slower in cold than warm environments (Maher 2010; Clark and Fritz 2013). Slower reaction rates in colder environments favor the production of immature waters. In contrast, faster reaction rates in warmer environments favor hydrochemically mature waters and the transformation of primary contaminants to secondary and tertiary forms.

Within the context of climate, geomorphology, as characterized by topography and the texture, thickness, and intensity of weathering of the regolith, controls hydrochemical maturity via infiltration, percolation, water storage, drainage, physical filtration (inc. straining), absorption/adsorption, chemical precipitation, dissolution, and acid neutralization processes (Fig. 1; Moldan and Cerny 1994; Maher 2010; Clark and Fritz 2013; Boyd 2019). Geomorphology, through its control of contact time, also influences the rate of microbially mediated redox processes, which directly or indirectly control the transformation, mobility, or attenuation of contaminants, and hence their susceptibility to loss (Moldan and Cerny 1994; Tratnyek et al. 2012; Burt et al. 2022).

Steeper slopes favor shallow flowpaths, overland flow and stormflow (i.e., flow at the contact between the base of the topsoil and the top of the subsoil or poorly permeable bedrock), and higher flow velocities, reducing the contact time with the underlying regolith, producing immature waters, even where infiltration capacities and hydraulic conductivities are favorable (Sterte et al. 2021; Burt et al. 2022). Conversely, gentler slopes can prolong contact time due to lower flow velocities, allowing more time for infiltration and subsequent matrix flow, producing hydrochemically more mature waters (Fig. 2). All other things being equal, steeper slopes are more susceptible to overland flow and stormflow, and the generation and loss of primary contaminants. Conversely, gentler slopes that favor infiltration, percolation, and matrix flow are more susceptible to the generation and loss of tertiary contaminants and exhibit lower susceptibilities to the loss of primary and secondary contaminant forms (e.g., Gao et al. 2020).

Regolith with low infiltration capacities or hydraulic conductivities is resistant to matrix flow and favors shallow hydrological flowpaths, leading to decreased contact time and the production of hydrochemically immature waters (i.e., low regolith-derived solute concentrations) (Fig. 2; Sterte et al. 2021; Burt et al. 2022). These regolith characteristics increase susceptibility to the generation and loss of primary and secondary contaminants via overland flow and stormflow (e.g., Fransen et al. 2023). Similarly, bedrock outcrops or shallow regolith, characterized by a low water-holding capacity, favor shallow hydrological flow paths and short contact times. These characteristics result in the production of hydrochemically immature waters and confer an increased susceptibility to primary and secondary contaminant loss.

Higher infiltration capacities and hydraulic conductivities favor deeper hydrological pathways, interflow and groundwater flow, hereafter “matrix flow,” resulting in longer contact times and the production of hydrochemically more mature waters (Sterte et al. 2021; Burt et al. 2022). The thicker the unimpeded regolith, the longer the path water must travel to a discharge point, resulting in greater contact times (Domenico and Schwartz 1998; Fetter 2018). Consequently, where infiltration of the pedolith and deeper matrix flow is favored, more mature waters are produced (McMahon and Chapelle 2008; Sterte et al. 2021; Burt et al. 2022), and susceptibility to the generation of tertiary contaminants increases (e.g., Di and Cameron 2002). Conversely, in the same setting, susceptibility to the loss of primary and secondary contaminants will be low due to greater filtering, straining, and absorption/adsorption rates. Where higher infiltration capacities and hydraulic conductivities favor matrix flow, susceptibility to tertiary nitrate is elevated. However, susceptibility to the loss of agriculturally derived tertiary P, i.e., orthophosphate, is typically low due to absorption/adsorption by the oxides and oxyhydroxides of iron, manganese, and aluminum, which are stable under the oxidizing conditions associated with high regolith infiltration capacities and hydraulic conductivities (McMahon and Chapelle 2008; Strawn et al. 2020).

For landscape units characterized by low infiltration capacities, low hydraulic conductivities, low water storage, or steep slopes, the majority of effective precipitation discharges to stream as overland flow or stormflow within minutes, hours, or days, producing hydrochemically immature waters (Inamdar 2011). Therefore, where overland flow and stormflow are favored, there is greater susceptibility to the generation and loss of primary and secondary contaminants and a lesser susceptibility to the generation and loss of tertiary contaminants (e.g., Fransen et al. 2023). Where overland flow is the dominant hydrological pathway, susceptibility to the loss of solid-phase fertilizers or animal wastes is elevated.

Landscape units that favor shallow subsurface stormflow or shallow interflow (e.g., shallow pedolith over bedrock or imperfectly to poorly drained pedolith that has been artificially drained) or have extreme infiltration capacities and hydraulic conductivities (e.g., coarse-textured alluvium, fracture-dominated systems, macropore bypass, or artificial drainage), produce waters of intermediate maturity and have a greater susceptibility to secondary, and in some settings, primary contaminant loss (Fig. 2; Austin et al. 2004; Schimel et al. 2007). For example, there is elevated susceptibility to the loss of primary contaminants, e.g., pathogens, where the regolith is characterized by significant macroporosity (e.g., shrink-swell soils, artificial subsurface drainage, fractured bedrock) or where alluvial regolith lacks a matrix of fines (i.e., clast supported gravels).

Within the context of contact time, the texture of the regolith also controls biogeochemical reactivity, with higher rates of reactivity for organic matter rich or fine (silt and clay) textured regolith relative to coarse textured (sand, granules, pebbles, cobbles, boulders) or organic matter poor regolith, due to greater surface areas and surface reactivities (Moldan and Cerny 1994; Fetter et al. 1999; Tan 2009). The reactive nature of fine textured regolith favors abiotic and biotic reactions that amplify hydrochemical maturation and the transformation of primary contaminants to secondary and tertiary forms (Tan 2009; Maher 2010).

Most landscape units exhibit temporal variation in the hydrochemical maturity of the waters they produce, in response to the time water spends in contact with the regolith (Inamdar 2011). The degree of temporal variation in the hydrochemical maturity of the waters produced by a landscape unit depends on its respective position along each of the climatic, geomorphic, and lithologic factor gradients. For example, given a broadly similar climate, temporal variation in the hydrochemical maturity of waters produced by a permeable, lowland alluvial plain landscape unit, is likely to be low relative to that produced by a deep, fine textured, albeit well drained, regolith associated with a hill country landscape unit. The permeable, lowland alluvial plain landscape unit will exhibit lesser temporal variation in hydrochemical maturity due to predominance by matrix flow and resulting discharge as groundwater flow. Whereas the deep, fine textured, well-drained regolith within a hill country unit, will exhibit greater temporal variation in hydrochemical maturity due to the production of waters via episodic runoff (overland flow and stormflow = immature), shallow interflow when the pedolith is saturated (intermediate maturity), and deeper interflow (mature) when the overlying pedolith is unsaturated.

Where hydrochemical maturity is strongly temporarily variable, susceptibility to the loss of different contaminant forms is also strongly temporarily variable (i.e., temporal production of primary through to tertiary contaminants). Conversely, in cases where hydrochemical variation shows minor temporal variability, we expect that temporal changes in susceptibility to the loss of various contaminant forms from a landscape will also be minor. Where temporal variation in hydrochemical maturity is low, susceptibility is often linked to a predominant or single contaminant form and predominant flowpath, such as nitrate-rich waters discharged as groundwater flow from a permeable, lowland alluvial plain landscape. Ultimately, temporal variation in the hydrochemical maturity of the waters produced, and the susceptibility of a landscape unit to contaminant loss is best defined as a continuum, with some landscape units exhibiting lesser and others greater temporal variation in the degree of the hydrochemical maturity of the waters produced and, consequently the susceptibility to the loss of the different contaminants forms.

Finally, we recognize that the composition of the regolith (i.e., its parent materials or “lithology”) is an important factor controlling the relative magnitude of mineralization, and hence the hydrochemical maturity, of the waters produced by a landscape unit (Güler and Thyne 2004; Robinson and Kapo 2003). However, because the PEC focuses on describing the landscape’s susceptibility to contaminant loss, we have not explicitly included consideration of the reactivity of different rock (e.g., basalt vs. granite) or sediment (e.g., peat vs. travertine) types on the relative abundances of the major ions (i.e., major ion facies). Rather, the PEC conceptualizes the factors controlling the contact time between precipitation and the regolith, irrespective of the specific reactivity of different rock or sediment types. Our rationale is that for the same rock or sediment type, a shorter contact time will produce immature waters, whereas a longer contact time will produce more mature waters. Consequently, the focus of Level 3 of PEC is on the lithological factors that control microbially mediated redox and the supply of fine sediment to streams, given the importance of the outcomes of these processes on water quality.

#### Factor categories, mapping, and characteristics

At each system level, variations in specific processes determining the hydrochemical maturity and susceptibility to contaminant loss are discriminated by subdividing factors into categories. PEC classes at each system level are defined by concatenating the controlling factor categories assigned to that unit for that and all preceding levels. Combining categories to produce PEC classes means hydrochemical maturity and susceptibility to contaminant loss are represented as the product of processes represented by each system level.

We separate the classification process (i.e., the characterization and labeling of categories) from the mapping process. The mapping process involves assignment, i.e., choosing or recognizing the category to which a landscape unit belongs; Klijn 1994). To make the process of mapping the PEC repeatable and transparent, the assignment is based on mapping characteristics and mapping rules (*sensu* Klijn and de Haes 1994). Mapping characteristics are continuous or categorical spatial data, preferably available as GIS coverages. The choice of mapping characteristics includes subjective judgments and may also be constrained by the availability of appropriate spatial data. However, mapping rules are unambiguous conditional statements that exhaustively assign all parts of the classification domain to a category based on the mapping characteristics. PEC classes at each level are defined by a top-down concatenation of the categories represented by each hierarchical level.

**Fig. 3:**
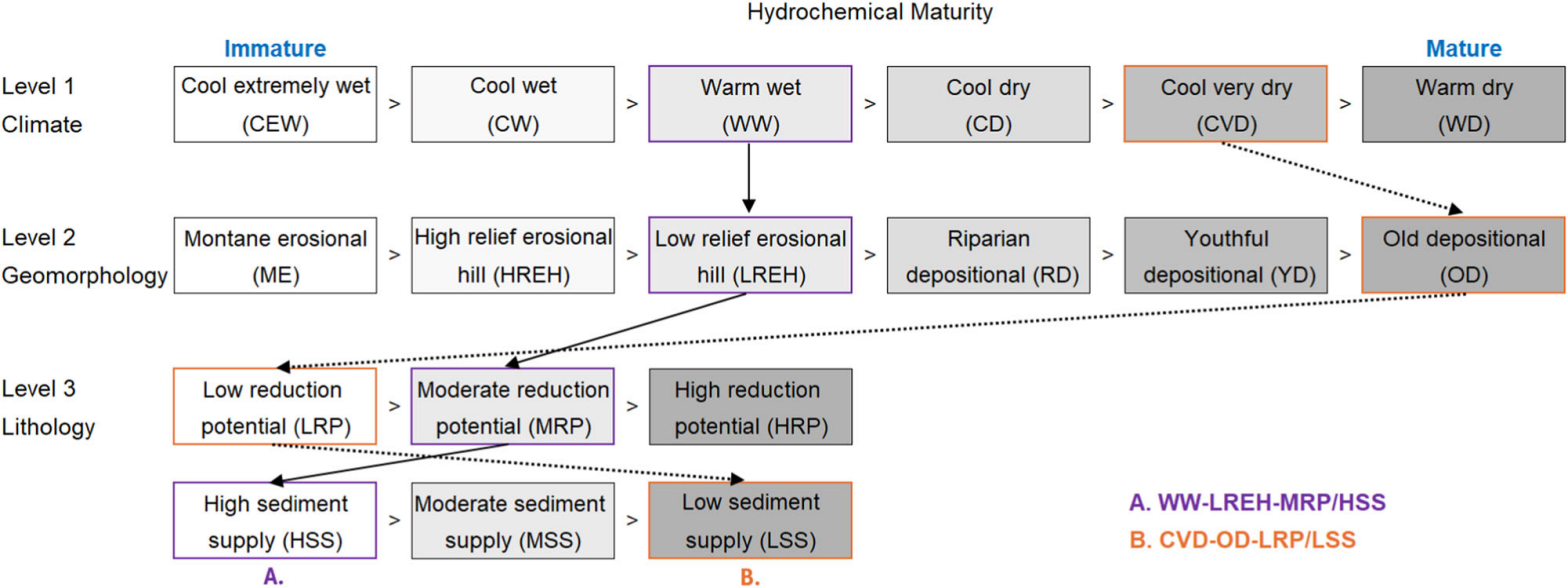
Physiographic Environment Classification categories at each system level. Hydrochemical maturity increases from left to right (light to dark). Arrows between categories at each level provide examples of the concatenation of categories to produce classes A and B (see also Fig. 6)

Expectations concerning the hydrochemical maturity of waters produced by a PEC class are obtained by ordering the categories at each system level, from least to most mature, based on an understanding of each category’s control over the magnitudes of the processes represented by that level (Fig. 3). Ordering can be facilitated by using selected mapping characteristics as surrogate measures of the relative magnitudes of the relevant processes. For example, climate categories at Level 1 can be ordered using mapping characteristics such as effective precipitation and air temperature. A climate category that is characterized by the highest and lowest values of these mapping characteristics, respectively (e.g., cool extremely wet climate category), is expected to produce the least hydrochemically mature waters due to the highest precipitation, lowest heating, highest cooling, and lowest evaporation rates. All other things being equal, this climate category is expected to produce the highest rates of porewater displacement, overland flow, and stormflow, the lowest evaporative concentration, and, consequently, the least hydrochemically mature waters. Conversely, a climate category characterized by the lowest and highest rates of effective precipitation and air temperature (e.g., warm dry climate category) is expected to produce the hydrochemically most mature waters.

The ordering of categories in terms of hydrochemical maturity is the basis for inferring the susceptibility of landscape units to the loss of primary, secondary, and tertiary contaminant forms. For example, at Level 3 (Lithology), a category of rock and sediment with low electron donor abundance is expected to produce waters characterized by a strongly positive oxidation-reduction-potential (ORP). A landscape unit that produces oxidizing waters is expected to have a high susceptibility to nitrate loss but a low susceptibility to the loss of dissolved P forms (dissolved organic and inorganic orthophosphate). Conversely, a category associated with a high reduction potential (high electron donor abundance and negative ORP) is expected to have a low susceptibility to nitrate loss but an increased susceptibility to the loss of organic and inorganic forms of P and the chemically reduced organic and inorganic forms of N.

The principle of hierarchical control over processes at each system level means that ordering categories at Levels 2 and 3 of PEC represent process maxima, the potential of which may or may not be realized depending on the constraints provided by the higher levels. An example of this is provided by a landscape unit assigned to a lithological category (Level 3) characterized by a high electron donor abundance. Depending on the control exerted by processes at higher system levels, this landscape unit may produce weakly reducing or oxidizing waters. Weakly reducing or oxidizing waters might occur if higher system levels meant little opportunity for microbially mediated redox reactions. A lack of opportunity could be due to climatic processes (Level 1; e.g., cold temperatures that inhibit microbial redox) or hydrological processes (Level 2; e.g., water runs off a poorly permeable, albeit electron donor-rich regolith instead of infiltrating into and percolating through the regolith before discharge). Thus, climatic and hydrological processes represented at the first and second levels of the PEC hierarchy constrain the magnitude of microbially mediated redox succession at the third and lowest system level.

This hierarchical control principle also applies to PEC’s characterization of the susceptibility of landscape units to contaminant loss. A PEC class characterizes susceptibility as a series of constraints on the rate of primary, secondary, and tertiary contaminant generation, retention, loss, and attenuation at each system level. This means that PEC may predict that the same or similar contaminant loss susceptibility can occur due to differing sets of processes. Elucidating the combinations of the processes responsible for the susceptibility of a landscape unit to contaminant loss provides important information to land and water managers. For example, elevated susceptibility to organic and ammoniacal-N losses may be due to either overland flow (steep slope or low infiltration capacity) or the drainage of strongly reduced groundwater from a shallow, organic carbon-rich aquifer to the stream network. Identifying the causal processes is critical for the effective mitigation of land use-derived contaminant loss, including an understanding of which aspects of contaminant loss are manageable (e.g., overland flow *may* be easier to mitigate than groundwater-derived losses from a reducing aquifer) or otherwise inherent to the landscape unit, e.g., low Dissolved Oxygen (D.O.), elevated organic N, ammoniacal N, and organic P concentrations in groundwater discharge may be a natural consequence of the aquifer composition and unrelated to land use (e.g., Price et al. 2023).

### Application to New Zealand

The subdivision of the controlling factors into categories for our application of the PEC to New Zealand is described (Fig. 3). The number of categories, their ordering in terms of hydrochemical maturity, and their expected susceptibilities to primary, secondary, and tertiary contaminant generation, retention, and loss are described below. Although our approach to categorization of the controlling factors would apply in other geographic settings, the number and character of the categories identified here are specific to New Zealand’s climatic, geomorphic, and lithologic range.

#### Level 1: Climate categories

Level 1 categories subdivide the landscape into climatically distinctive landscape units at a characteristic scale of 10^5^ – 10^4 ^km^2^ (macroscale). Within the context of lower levels, we propose that climate controls variation in processes at the macroscale, including precipitation, heating/cooling, and evaporation (Fig. 1). All other things being equal, we expect effective precipitation to control the time water spends in contact with the regolith via episodic displacement of pore waters and the activation of surficial flow paths (e.g., overland flow).

In combination with precipitation, we expect temperature will control abiotic and biotic reaction rates with higher rates of organic matter accumulation and microbial transformations (e.g., redox) in warm, humid climates relative to cold or dry climates. Further, we expect pore waters exposed to high evaporative concentration rates to be more mature than the converse. We expect the climate to control the intensity of rainfall, influencing the per unit time kinetic energy of the raindrops and their capacity to detach and mobilize pedolith or entrain surficial debris.

We propose six climate categories specific to New Zealand’s climatic range: Level 1, Cool Extremely Wet (CEW); Cool Wet (CW), Warm Wet (WW), Cool Dry (CD), Cool Very Dry (CVD), and Warm Dry (WD). Ignoring lower system levels, we anticipate that the hydrochemical maturity of the waters produced by these climate categories will progressively increase, following this sequence: CEW < CW < WW < CD < CVD < WD (Fig. 3). In this sequence, and again disregarding lower levels, we expect a decline in the susceptibility of primary contaminants to generation and loss, in contrast to an increase in the generation and loss susceptibility of tertiary contaminants. However, the susceptibility of secondary contaminants is expected to exhibit a more complex, approximately U-shaped trend across the hydrochemical maturity sequence.

Subdivisions of latitude and topography, described by broadscale maps, could be used to map climatic categories (e.g., Bailey 1998). Alternatively, mapping characteristics can be spatial coverages of climate summaries, such as effective precipitation and air temperature. Climate categories can be ordered using mapping characteristics, such as effective precipitation and temperature, to differentiate differences in the expected magnitude of pore water displacement, overland flow, and evaporative concentration.

#### Level 2: Geomorphic categories

Level 2 categories subdivide the landscape into geomorphically distinctive landscape units with a characteristic scale of 10^4^–10^2 ^km^2^ (mesoscale). Within the context of other levels, we propose that physical geomorphology determines the hydrochemical maturity of the waters produced by a landscape unit by determining the hydrological pathways water takes across or through the regolith by controlling rates of infiltration, percolation, drainage, and regolith storage. Within the context of climate, we propose that geomorphology further discriminates contact time and, therefore, influences the time-bound component associated with the generation, transformation, retention, transport, and attenuation of agricultural contaminants. This influence occurs through a variety of mechanisms, including but not limited to mobilization, filtration, straining, adsorption, mineral dissolution, chemical precipitation, acid neutralization, and microbially mediated redox processes. Six geomorphic categories specific to New Zealand’s geomorphic range are proposed (SI 1 contains a graphic to support the following text).

We propose that the dominant hydrological flowpaths vary from surficial (e.g., overland flow) to deep matrix flow (e.g., groundwater flow from the portion of the water table aquifer that is highly coupled to stream), and contact times increase across a geomorphological gradient from erosional (mountains, hills) to depositional (lowland plains, deltas) and from supply to transport limited slopes. Therefore, to discriminate hydrological flow path and contact time, we propose subdividing the landscape into erosional and depositional geomorphic categories and further subdividing the erosional geomorphic categories into three categories that distinguish supply, mixed supply–transport, and transport limited landforms.

We characterize the Montane Erosional (ME) category as supply limited, where sediment export rates exceed sediment accumulation rates, resulting in large areas of bare rock and incipient (rocky or thin) regolith. We characterize High Relief Erosional Hill (HREH) as having a mix of supply and transport-limited landforms, with steep slopes associated with supply limitation and gentler slopes, often at lower elevations, with transport limitation. The Low-Relief Erosional Hill (LREH) category is characterized as transport-limited, with sediment accumulation rates exceeding the sediment export rate, at least in the prehistoric, resulting in the accumulation of a deep mantle of fine textured regolith.

Therefore, within the context of other levels, we propose that infiltration and matrix flow increases as relief decreases and the abundance of fine-textured materials and regolith thickness increases across the ME (Supply Limited) < HREH (Supply - Transport Limited) < LREH (Transport Limited) erosional geomorphic continuum. At the same time, we expect that the contact time and the resultant hydrochemical maturity of the waters produced increases across the same sequence. Because of the opportunity for deep flowpaths within the transport-limited LREH category, we expect a significant range in the hydrochemical maturity of the water produced with discharge volume (i.e., variation through immature (high volume), intermediate, and mature (low volume)), and, as a result, we expect these landforms to exhibit greater susceptibility to the generation and loss of significant quantities of all three contaminant forms relative to the ME and to a lesser degree HREH categories.

Within the ME category, due to the combination of high-relief, large areas of bare rock and shallow, coarse-textured regolith, we expect contact times to be the shortest of all geomorphic categories. Due to the shortest contact times and disregarding other levels, we expect the waters produced by the ME category to be the least hydrochemically mature of all the geomorphic categories (lowest regolith-derived solute concentrations). As a result of the shortest contact times and the absence of deep, fine-textured regolith for enrichment by agricultural contaminants or disturbance mechanically, we expect the production of primary, secondary, and tertiary contaminants to be the lowest of all the geomorphic categories. However, we expect ME units to be highly susceptible to losing any primary and secondary contaminants introduced at the land surface by agricultural activities.

We characterize landscape units belonging to the LREH geomorphic category as having a thick blanket of fine textured and slowly permeable regolith that produces large volumes of overland flow, stormflow, and lesser, albeit ecologically important, volumes of interflow. Within LREH units developed for agriculture, the fine-textured regolith is expected to have a high capacity for retaining agricultural contaminants and greater susceptibility to mechanical disturbance when contrasted with the ME category. Consequently, within the context of other levels, enriched LREH regolith is considered the most susceptible of the geomorphic categories to generating and losing both primary and secondary contaminants.

Conversely, outside of high-intensity rainfall events, we propose that slow infiltration, percolation, and the generation of interflow are associated with greater matrix flow and, hence, contact times for the LREH relative to the ME and HREH categories. Within the context of other levels, we expect this to result in an increased susceptibility of the LREH category to the generation, retention, or loss of tertiary contaminants and the production of moderate to mature waters where interflow pathways are sufficiently long or deep. However, relative to overland flow and stormflow, we expect that the volume of water and the total (all contaminants) per unit load of contaminants produced by interflow will be small. Therefore, within the context of other levels, we expect LREH units to be highly susceptible to primary and secondary contaminant generation and loss, with moderate susceptibility to the generation and loss of tertiary contaminants via interflow.

Contact times within the HREH category are considered transitional between ME and LREH due to the presence of both supply and transport-limited slopes. Within the context of other levels, we propose that higher organic matter accumulation rates and greater fine sediment abundance within HREH equate to greater susceptibility to primary and secondary contaminant generation and loss relative to ME. Relative to ME, longer contact times are expected to favor greater generation and potential for loss of tertiary contaminants, with a broadly equivalent susceptibility to LREH, where deeper and more permeable regolith occurs (e.g., transport-limited slopes that occur at lower elevations).

We propose three depositional geomorphic categories, all of which are transport-limited: Riparian Depositional (RD), Youthful Depositional (YD), and Old Depositional (OD). Within the context of other levels, we expect matrix flow to dominate within depositional landforms, with greater contact times relative to the erosional landforms. We expect that contact times and hydrochemical maturity will increase across the following sequence, RD < YD < OD, in response to decreasing infiltration capacities and hydraulic conductivities due to age and attendant weathering-related increases in compaction, secondary mineral formation, and organic matter accumulation (Fig. 3). Consequently, within the context of other levels, we expect decreasing susceptibility to secondary contaminant loss and increasing susceptibility to the generation of tertiary contaminants across the depositional sequence. An increase in susceptibility to primary and secondary contaminant loss for fine textured regolith across depositional geomorphic categories is characterized at Level 3 (“Level 3 Lithology”).

The RD is characterized as the youngest of the geomorphic categories. It is associated with the “modern-day” (i.e., within the last 1000 years before the present (ky BP)) floodplain of high-volume and high-energy river systems with headwaters within high-relief ME or HREH categories. Due to the youth of the floodplain, we expect the regolith to be coarse-textured, with the greatest infiltration capacities (>70 mm/h) and regolith hydraulic conductivities (10^−4^ to 1 meter per second (m/s)) (e.g., Sophocleous 2002) of all the geomorphic categories. In contrast, we consider the stream power of low-energy or low-volume river systems, i.e., those rivers that do not have headwaters in large ME or HREH catchments, insufficient to generate coarse-textured and highly transmissive riparian regolith.

Within the context of other levels, we expect that due to the relatively low abundance of fine textured sediments, low water storage rates, high infiltration capacities, and hydraulic conductivities, the RD category has the greatest susceptibility of all the geomorphic categories to the generation and loss of secondary contaminants. In contrast, the susceptibility of the RD category to the generation of tertiary contaminants is regarded as low relative to the YD and OD categories. If flooding drives water tables to the surface, we expect an elevated susceptibility to the entrainment of land-use-derived primary contaminants as overland flows directly to the drainage network. Due to dilution by ME or HREH-sourced river waters, regolith-derived solutes, primary, secondary, or tertiary contaminants that reach the water table aquifer hosted by RD are rapidly diluted. Irrespective of the contaminant type lost to the water table, the RD units’ coarse texture and low water holding capacity equates to shorter contact times and lesser opportunity for attenuation of agriculturally derived contaminants relative to the YD and OD categories.

We categorize landscape units as YD if deposited after the last late glacial maxima, mainly between 1 and 14 ky BP. We define YD landscape units as derived from sediment exported from high-elevation landforms (ME and HREH categories), including ejecta (e.g., volcanic ash and ignimbrite deposits) from large-scale rhyolitic or andesitic volcanic systems. Landscape units classified as YD are expected to host significant shallow aquifer systems (Rosen and White 2001). Due to their youthfulness and proximity to ME and HREH, YD units are expected to retain subsurface hydrogeological connections to high-elevation recharge areas with subsurface inflows of immature waters constituting a volumetrically important source of groundwater recharge (e.g., Rissmann et al. 2015).

As a result of their youthfulness and proximity to sediment source areas, units classified as YD are characterized by thicker deposits of coarse-textured materials (boulders, cobbles, pebbles, granules, or sands), with a greater abundance of silt and clay relative to the RD category. Due to greater infiltration capacities and hydraulic conductivities, most rainfall is expected to infiltrate YD units, moving through the unsaturated and saturated matrix of the regolith. Therefore, where flat, we expect YD categories to exhibit the lowest incidence of overland flow and stormflow of the geomorphic categories and, consequently, the lowest susceptibility of all the categories to primary and secondary contaminant loss.

Due to dominance by land surface recharge and longer contact times, we expect YD units to exhibit greater susceptibility to the generation and loss of nitrate and its retention within shallow water table aquifers relative to the RD geomorphic category. However, nitrate concentrations in water table aquifers of the YD unit may be variable, with lower concentrations for an equivalent land use intensity, where aquifers are diluted by the throughflow of high-elevation recharge produced by ME or HREH units. With regards to tertiary contaminants, the YD category is expected to be more susceptible to the generation and retention of agricultural orthophosphate than the ME, HREH, and RD geomorphic categories, yet less susceptible to its loss via leaching due to greater contact times and larger surface areas, that favor P-retention. Despite a low susceptibility to leaching of agriculturally derived orthophosphate, the groundwater flow produced by YD units is expected to contain elevated concentrations of geogenic-P, also as orthophosphate, due to the dissolution of mineral P (e.g., apatite) along the flowpath (e.g., Tao et al. 2020).

We categorize landscape units as OD if deposited during or before the last glacial maximum, mainly between 29 and 428 ky BP. These landscape units occupy lower elevation areas, commonly large coastal plains or inland basins, having been deposited by glacial processes, ancestral river systems, or volcanism. They are characterized by the most weathered regolith of the geomorphic categories. Due to their greater age, we propose that OD are characterized by lower infiltration capacities and hydraulic conductivities than RD or YD, which we consider a factor of weathering-induced increases in organic matter, silt, and clay abundances. We propose that due to their age-related position in the landscape, they seldom retain a subsurface connection to high-elevation source areas (ME and HREH) and that due to lower hydraulic conductivities, their groundwater systems are not highly coupled to adjacent, younger (RD or YD) landscape units. Consequently, precipitation deposited at the land surface is considered the dominant recharge source, with negligible dilution of groundwaters by ME or HREH-derived waters.

Contact times peak within the OD category, relative to RD and YD, due to dominance by land surface recharge, lower infiltration capacities, hydraulic conductivities, and matrix flow. Therefore, within the context of other levels, the OD category is regarded as producing the most mature waters and as being the most susceptible to the accumulation of leached nitrate (>6 mg/L NO_3_-N) within the shallow water table aquifer. Conversely, within the context of other levels, although land-use-derived orthophosphate is expected to accumulate to high levels within the pedolith, its susceptibility to leaching is considered the lowest of all geomorphic categories due to age-related increases in secondary clay mineral production (including the oxides and oxyhydroxides of iron, manganese, and aluminum), which favors P-absorption/adsorption. Further, we propose that due to the greater weathering of OD units, the waters they produce will contain lower concentrations of geogenic-P than RD and YD landscape units. An increase in susceptibility to primary and secondary contaminant loss for fine textured regolith within the OD geomorphic category is characterized at Level 3 (“Level 3 Lithology”).

Disregarding the influence of higher and lower system levels, we propose that the maturity of waters produced by Level 2 geomorphic categories increases across the following sequence: ME (immature) < HREH < LREH < RD < YD < OD (mature) (Fig. 3). Across this sequence, we expect an increase in contact times and susceptibility to nitrate generation and its loss to surface waters as interflow or shallow groundwater flow. Consequently, nitrate concentrations within shallow groundwaters are expected to increase across the RD < YD < OD sequence.

Overall, we expect susceptibility to the loss of primary contaminants to increase from ME to LREH, with ME having the lowest and LREH the highest susceptibility in this sequence (ME < HREH < LREH). This trend then reverses, with a sharp decrease in susceptibility for the RD (except where gravels are clast supported), and especially the YD category. The OD category’s susceptibility to primary contaminant loss is expected to increase sharply due to weathering-related decreases in infiltration capacity and hydraulic conductivity, favoring greater overland flow or artificially mediated stormflow and interflow, especially where the pedolith is fine textured (“Level 3 Lithology”). With regards to secondary contaminants, we expect that susceptibility increases progressively from ME to LREH (ME < HREH < LREH), reaching a peak within the RD category, before decreasing sharply for the YD, followed by a significant increase for the OD category, especially where the pedolith of the latter is characterized by low infiltration capacities or significant macroporosity (e.g., shrink-swell soils or artificial subsurface drainage).

Overall, we expect susceptibility to the generation and retention of agricultural orthophosphate to peak within the pedolith of the OD, LREH, and, to a lesser degree, the YD categories due to greater surface areas and abundances of P-retaining clay minerals. However, due to high retention rates, we expect susceptibility to the leaching of agricultural orthophosphate to be lowest for the YD, LREH, OD, and the transport-limited slopes of the HREH category. Although the susceptibility of YD units to leaching of agriculturally derived orthophosphate is considered low, we expect discharging waters to contain elevated concentrations of naturally derived geogenic P. The generation and retention of tertiary orthophosphate is expected to be lowest for the ME, RD, and supply-limited slopes of the HREH category. An increased susceptibility to the leaching of agricultural orthophosphate due to lithological characteristics is characterized at Level 3 (“Level 3 Lithology”). Similarly, a decrease in the susceptibility to nitrate leaching due to microbially mediated redox processes, i.e., denitrification, is also discussed.

Subdivisions of geology and topography, described by broadscale maps, can be used to map geomorphic categories (e.g., Winter 2001). Geomorphic categories should be ordered according to the time water spends in contact with the regolith and, hence, the hydrochemical maturity of the waters produced, using mapping characteristics such as elevation, rock/sediment type, and geomorphic surface age.

#### Level 3 Lithology

Level 3 categories subdivide the landscape into lithologically distinctive landscape units with a characteristic scale of 10^2^–0.5 km^2^ (microscale). Within the context of higher levels, we propose that lithology controls variation in processes, including microbially mediated redox and sediment supply, which are important determinants of spatial variability in the susceptibility of landscape units to the generation, retention, loss, or attenuation of primary, secondary, or tertiary contaminants (Figs. 1 and 3). We define two sets of categories at Level 3, comprising Reduction Potential (RP) and Sediment Supply (SS), each of which subdivides the landscape into three categories (Low, Medium, and High).

Within the context of higher levels, we propose that lithology determines the reduction potential of landscape units by controlling the abundance and bioavailability of organic carbon and ferrous iron (electron donors) that fuel the microbially mediated succession of terminal electron-accepting processes within the regolith. We note that electron donors or electron acceptors hosted by strongly cemented rock or sediment may not be available to participate in microbially mediated redox reactions (e.g., Cutting et al. 2009).

The concept of microbial-mediated redox succession in freshwaters involves the sequential change in dominant microbial processes, which is determined by the availability of different electron acceptors (e.g., O_2_, NO_3_, Mn^IV^, Fe^III^), leading to changes in biogeochemical conditions and the persistence, form, and mobility of nitrogen and phosphorus species. We note that microbially mediated redox succession is important to the hydrochemical maturation of freshwater, the formation and enhanced mobility of nanometer scale colloidal and dissolved forms of phosphorus, the concentration of reduced forms of nitrogen (i.e., organic and ammoniacal N), and the attenuation of nitrite and nitrate via denitrification (Tratnyek et al. 2012 and others).

As redox succession advances, CO_2_ is liberated, and its partial pressure increases, increasing the alkalinity of water (if water is at or above a pH of 4.4). When succession progresses beyond Mn^IV^- or Fe^III^-reduction, dissolved Mn^II^ and Fe^II^ concentrations also typically increase (Tratnyek et al. 2012). Due to greater alkalinity, Mn^II^, or Fe^II^ concentrations, we consider reducing waters to be more hydrochemically mature than oxidizing waters. Therefore, within the context of higher system levels, we expect the hydrochemical maturity of the waters produced by RP categories to increase as follows: Low RP (immature) < Moderate RP < High RP (mature) (Fig. 3). Across this sequence, we expect susceptibility to the generation, leaching, or build-up of nitrate within the regolith (e.g., pedolith and water table aquifer) to decrease. In contrast, we expect the susceptibility to the generation, loss, and build-up of primary (particulate organic N and P) and secondary (dissolved organic-P and ammoniacal N), contaminants within the regolith to increase. If terminal electron-accepting processes move beyond Mn^IV^ and especially Fe^III^ reduction, we expect that the dissolution of P-retaining oxides and oxyhydroxides will result in an increased susceptibility to the leaching of agriculturally derived organic-P and orthophosphate. Broadscale lithology maps can be used to categorize and map RP categories according to the abundance and bioavailability of electron donors (e.g., Krantz and Powars 2002).

We propose that lithology controls the abundance of clay, silt, and organic matter (hereafter, “fine sediment”) available for supply. All other things being equal, we expect coarsely-textured rock/sediment to yield less fine- sediment than fine-textured rock/sediment and weakly-weathered rock/sediment to yield less fine sediment than strongly-weathered rock/sediment (e.g., Whipple and Tucker 1999). Regolith formed in organic sediment is expected to yield more particulate organic matter than regolith formed in inorganic sediments (e.g., Marttila and Kløve 2015).

We also expect clays, silts, and organic matter to be associated with greater abundances of surface absorbed/adsorbed and structural nitrogen or phosphorus, especially where the land has been developed for agricultural uses (e.g., Brady and Weil 2008). Therefore, we reason that where land has been developed for productive purposes, finely textured regolith will contain significantly greater quantities of labile carbon (i.e., lower C:N and C:P ratios), N, P, and pathogens per unit than coarsely textured regolith. We also expect fine-textured regolith to exhibit greater susceptibility to mechanical detachment in response to livestock movements or cultivation than coarse-textured regolith.

Within the context of higher levels, we expect that the lower infiltration capacities and hydraulic conductivities of landscape units assigned to the Moderate and High SS categories will be correlated with greater rates of overland flow and stormflow than the Low SS category. We expect overland flow and stormflow to enhance detachment and mobilization of enriched pedolith, increasing susceptibility to the loss of primary and secondary contaminants. Due to the greater per unit load of agricultural contaminants produced by fine-textured vs. coarse-textured regolith, we expect the streams dominated by overland flow and stormflow produced by M and H SS categories to exhibit evidence of greater internal eutrophication. Greater internal eutrophication relates to the smothering of benthic habitat by fine sediment and subsequent sediment anoxia related to increased loading of labile carbon (low C:N and C:P ratios). Furthermore, in response to the reductive dissolution of mineral oxides and oxyhydroxides and the oxidation of labile organic carbon, agriculturally enriched bed sediments are expected to drive the release of organic-N, ammoniacal-N, and organic and inorganic-P into the overlying water column over the long term (Stutter et al. 2018).

Where M and H SS categories have been artificially drained, we expect a decrease in overland flow in response to low/moderate-intensity rainfall events but an increase in susceptibility to the loss of secondary and tertiary contaminants via the artificial drainage network (e.g., Skaggs et al. 1994). Where developed for agricultural use, Level 2 depositional and LREH categories that are characterized by fine textured pedolith or pedolith that is underlain at relatively shallow levels by poorly permeable saprolith or saprock, i.e., M and especially H SS categories, are more likely to produce reducing waters due to lower aeration (e.g., mottling or gleying, iron pan formation). Therefore, within the context of the reduction potential category, we expect M and H SS categories to exhibit a lower susceptibility to nitrate leaching losses but greater susceptibilities to the leaching of agriculturally derived orthophosphate, secondary organic-P, organic-N, and ammoniacal-N than the L SS category.

Lithological SS categories are ordered according to the expected maturity of the waters they produce, i.e., from Low SS (mature) < Moderate SS < to High SS (immature) (Fig. 3). Broadscale lithology maps can be used to categorize and map SS categories according to the abundance of fine sediment within the regolith.

### Classification Process

**Fig. 4:**
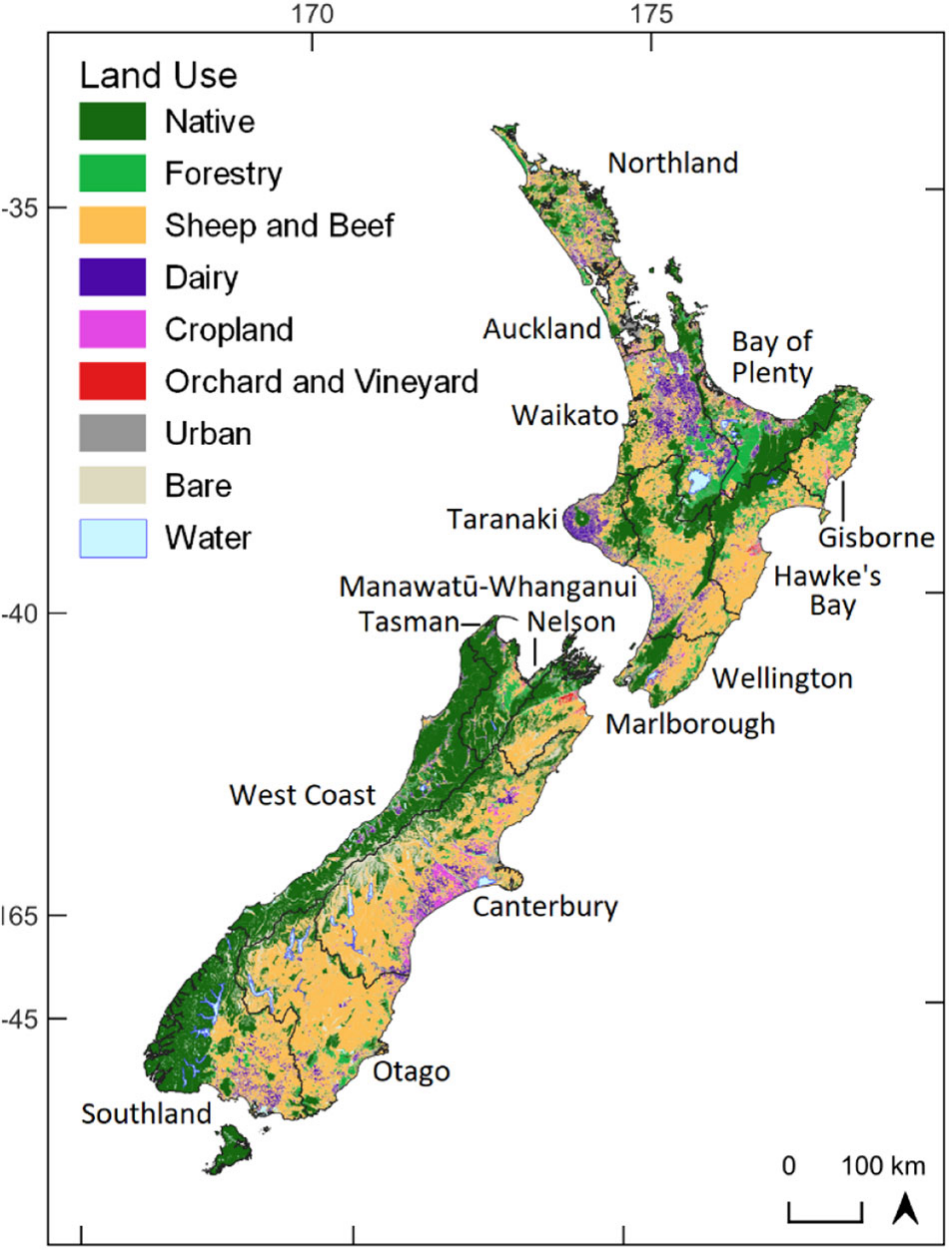
Map of the classification domain comprising New Zealand (268,021 km^2^) showing geopolitical regions and land use

We applied the PEC approach to classifying all land in New Zealand (268,021 km^2^) using available spatial data (Fig. 4 and Table 1). Each factor was represented by several geospatial layers (i.e., mapping characteristics). Each landscape unit was then assigned to factor categories at each level of the PEC hierarchy by applying the rules to the relevant geospatial layers within a GIS.

**Table 1:** Factors, mapping characteristics, data sources, and ordering of PEC categories according to hydrochemical maturity

Factor	Mapping characteristics	Data source	Order of categories according to the hydrochemical maturity of the water produced (Least to most mature)
Level 1: Climate	Mean annual air temperature and mean annual effective precipitation.	Land Environments of New Zealand (LENZ 2009).	Cool Extremely Wet (CEW; immature) < Cool Wet (CW) < Warm Wet (WW) < Cool Dry (CD) < Cool Very Dry (CVD) < Warm Dry (WD; mature).
Level 2: Geomorphology	Landform (erosional vs. depositional), rock and sediment, geomorphic surface age, topography (elevation and slope), and hydrological connectivity to high-volume river systems.	Geological Survey (Q-Map v. 3: 1:250,000; Heron 2020); River Environment Classification and Digital River Network (v. 2.4) of Snelder and Biggs (2002) and the MERIT Digital Elevation Model (13 m) of Yamazaki et al. (2017).	Montane Erosional (ME) (immature) < High Relief Erosional Hill (HREH) < Low Relief Erosional Hill (LREH) < Riparian Depositional (RD) < Youthful Depositional (YD) < Old Depositional (OD) (mature).
Level 3: Lithology	Rock/sediment type, rock strength, electron donor abundance (organic matter and ferrous iron (Fe^II^) abundance), fine sediment (silt and clay), and organic matter content.	Geological Survey (Q-Map: 1:250,000; Heron 2020)	Reduction Potential (RP): Low (immature) < Moderate < High (mature). Sediment Supply (SS): High (immature) < Moderate < Low (mature).

#### Mapping rules

##### Level 1: Climatic categories

Maps of effective precipitation and air temperature were subdivided to define six climate categories (Table 1). Mean annual air temperature was subdivided into Warm (≥12 °C) and Cool (<12 °C) categories. Mean annual effective precipitation was subdivided into Extremely wet (≥1500 mm; South Island only), Wet (500–1500 mm).

Dry (0–500 mm) and Very Dry (≤0 mm; South Island only) categories. Effective precipitation categories subdivided air temperature categories to generate six climate categories, which we ordered from highest to lowest effective precipitation and lowest to highest temperature to discriminate variation in the expected hydrochemical maturity of the waters they would produce (Table 1).

##### Level 2: Geomorphic categories

Categories of rock/sediment type that appear on the national geological survey were assigned to the “Erosional (bedrock)” or “Depositional (non-bedrock)” categories (Table 1). A “Riparian Depositional” category was isolated from the “Depositional” category after filtering to exclude all rock/sediment types other than those designated as “modern-day floodplain” by geological survey. A digital river network was filtered to exclude all river networks that were smaller than 6th order at their terminal reach, and the resultant river network intersected with the “Depositional” categories identified as “modern-day floodplain” and the resulting landscape units classified as “Riparian Depositional.” The “Riparian Depositional” and the larger area of landscape units classified as “Depositional” were assigned geomorphic surface age categories according to national geological survey and expert knowledge. The categories were named Riparian Depositional (RD; modern-day floodplain; geomorphic age c. modern day to 1,000 ky BP), Young Depositional (YD; geomorphic age c. 5 to 14 ky BP), and Old Depositional (OD; geomorphic age c. 29 to 428 ky, oldest). The remaining landscape units, those classified as “Erosional,” were subdivided by elevation and assigned to relief categories: Montane Erosional (ME) > 800 m RSL; High Relief Erosional Hill (HREH) 300 ≤ 800 m RSL, and Low Relief Erosional Hill (LREH), <300 m RSL. The categories were then ordered according to the expected hydrochemical maturity of the waters they produce (Table 1).

##### Level 3: Lithological categories

*Reduction potential categories:* Categories of rock/sediment type recorded by national geological survey were assigned to “Consolidated (bedrock)” and “Unconsolidated (non-bedrock)” categories (Table 1). The “Consolidated” category was subdivided into low, moderate, or high rock strength categories using Hoek and Brown’s (1997) framework. The “Consolidated” category was subdivided into low, moderate, or high categories according to the abundance of organic matter and ferrous iron recorded by geological survey. The rock strength and electron donor abundance categories of “Consolidated” rock/sediment were subsequently concatenated (e.g., Low Strength-High Electron Donor Abundance, Low Strength-Low Electron Donor Abundance, High Strength-Low Electron Donor Abundance), and the groups assigned to “Low,” “Moderate,” or “High” Reduction Potential (RP) categories. The electron donor abundance of “Unconsolidated” categories was subdivided into Low, Moderate, or High RP categories according to the abundance of electron donors. The RP categories were then ordered according to the expected hydrochemical maturity of the waters they produce (Table 1).

*Sediment supply categories:* Categories of rock/sediment type recorded by national geological survey were assigned to “Consolidated (bedrock)” and “Unconsolidated (non-bedrock)” categories (Table 1). The “Consolidated” category was subdivided into low, moderate, or high rock strength categories using the framework of Hoek and Brown (1997). The “Consolidated” category was subdivided into low, moderate, or high categories according to the abundance of clay and silt-sized grains or the abundance of organic matter (e.g., coal), hereafter “fine sediment,” by applying the petrological grain size assessment framework of Blatt et al. (2006) to geological survey. The rock strength and fine sediment abundance categories of “Consolidated” rock/sediment were subsequently concatenated (e.g., Low Strength - High Fine Sediment Abundance, Low Strength - Low Fine Sediment Abundance, High Strength- Low Fine Sediment Abundance), and the resultant groups assigned to “Low,” “Moderate,” or “High” Sediment Supply (SS) categories. The “Unconsolidated” category was assigned to “Low,” “Moderate,” or “High” SS categories using the description of grain size recorded by geological survey. The SS categories were then ordered according to the expected hydrochemical maturity of the waters they produce (Table 1).

### Evaluation of the Classification Using Long-Term Monitoring Data

#### Water quality data

Median values of monthly observations of six water quality indicators (hereafter, water quality variables, Table 2) for the 2014–2018 period were compiled for 885 long-term surface water monitoring stations using the Land Air Water Aotearoa (LAWA) national water quality monitoring dataset (minimum 60 observations per station; Milne et al. 2019; SI 2). We filtered the observations to remove extreme outliers using Tukey’s Quantile Outlier method (1977). Extreme outliers are observations 3x the interquartile range past the first and third quartiles. Most extreme outliers were associated with stations occurring within geothermally active regions and those influenced by tidal incursions of seawater. Seventy-five sites were removed from the original dataset, leaving 810 stations.

**Table 2:** Water quality variables used in this study

Name	Symbol	Units	Description	Type	Contaminant Type
Turbidity	Turb	NTU^a^	Optical indicator of water clarity	Field measure	Primary
*Escherichia coli*	*E. coli*	cfu/100 mL	Indicator of pathogen contamination	Laboratory measure	Primary
Particulate Phosphorus	PP	mg P/L	Inorganic and organic forms of phosphorus >0.45 μm	Laboratory measure	Primary
Total Kjeldahl Nitrogen	TKN	mg N/L	Organic-N and ammoniacal-N	Laboratory measure	Secondary
Nitrate-Nitrite-Nitrogen	NNN	mg N/L	Nitrate and nitrite oxyanions	Laboratory measure	Secondary/Tertiary
Dissolved Phosphorus (reactive)	Dissolved-P (DRP)	mg P/L	Inorganic and organic forms of phosphorus <0.45 μm	Laboratory measure	Secondary/Tertiary

^a^Nephelometric turbidity unit

The proportions of catchment area occupied by each PEC category and class were calculated for each water quality station. To isolate the effect of individual PEC categories and classes, we included only those sites for which the dominant PEC class occupied ≥75% of the catchment area (the occupancy criteria). To ensure a minimum level of representation, we also required at least four monitoring stations to be assigned to each category or class in the following analyses. Due to small occupancy values, landscape units classified as RD could not be tested. All statistical tests were performed in R (R Core Team 2023), and the Variance Partitioning (VP) analyses used the “vegan” package.

#### Statistical tests

##### Variance partitioning

We assessed the overall performance of the PEC by quantifying the variation in the water quality variables explained by each level of the PEC. We recognized that a component of the variation in the water quality variables would be associated with land use, and, in addition, land use would co-vary with PEC classes. We used variance partitioning (VP) analysis (Borcard et al. 1992) to quantify the strength of relationships between the water quality variables and PEC classes while evaluating the extent to which these relations may be overestimated if land use was not accounted for.

The VP analysis used a series of multivariate linear regression models to partition the total variation in the water quality variables explained (i.e., R^2^) by two tables of explanatory variables representing land use and PEC classes. Land use was represented by the proportion of catchment area of each monitoring station occupied by the nine land use categories shown in Fig. 4. Only eight of the nine land use categories were included in the land use table because the variables are proportions that sum to one. Therefore, one of the variables was redundant. PEC classes were represented by a table of dummy variables indicating which PEC class each site was assigned. At each level of the PEC, the class tables had one less variable than the total number of classes to avoid redundant variables. A log (base 10) transformation was applied to the water quality variables to approximately normalize their distributions.

The variation explained by land use and PEC classes was partitioned into six components, including the total variation explained and the individual, shared, and unique contributions associated with land use and the PEC. A permutation test evaluated the significance of all components. The significance of the unique contributions was tested by running the complementary set of variables as co-variables (i.e., their effect was removed). Estimates of explained variation derived from samples are generally biased (Zar 1999). The number of independent variables in the model and sample size influence this bias. The method of Peres-Neto et al. (2006) was used to adjust the estimate of variation explained by each set of variables to make valid comparisons between sets of variables of differing sizes.

##### Analysis of variance

To increase the number of sites included in the analysis, we ignored PEC Level 1 categories and assessed the effect of PEC categories on the water quality variables at Level 2 (Geomorphology) using analysis of variance (ANOVA). We also applied ANOVA to assess the effect of PEC classes from concatenating Level 2 geomorphic and Level 3 (Lithology) SS categories on water quality. However, we could only include sites categorized as YD and OD at Level 2 due to limited monitoring station numbers, so we removed the occupancy criteria in order to have sufficient stations to evaluate Level 3 RP. Removing the occupancy criteria for Level 3 RP categories resulted in a minimum occupancy value of 41% (mean 84% and median 94%). We expected that the water quality variables would vary significantly between the PEC categories and classes and that their within-category mean values would vary according to the expected susceptibilities outlined in “Application to New Zealand”.

We removed the component of the variation in the water quality variables associated with land use in two steps. First, for each water quality variable, we fitted a linear regression model that is expressed mathematically as:

$${\log }_{10}\left(Y\right)={\beta }_{1}{P}_{1}+{\beta }_{2}{P}_{2}+{\beta }_{3}{P}_{3}+\ldots {\beta }_{8}{P}_{8}$$

where $Y$ represents the water quality values for each monitoring station, *P* is the proportions of the catchment of each monitoring station occupied by one of eight land use categories (i.e., excluding one redundant category), and ${\beta }_{1},{\beta }_{2},{\beta }_{3}\ldots {\beta }_{8}$ are fitted regression coefficients. Second, we retained the residuals of each regression model and assumed these represent the variation in the water quality variable that was not explained by land use.

We used Welch’s ANOVA, which is robust to unequal variances (Celik 2022) and reported the *R*^*2*^ and p-values for each test. We assessed the consistency of the water quality variables within each category with expectations associated with the conceptual model explained in “Application to New Zealand” by plotting the data as box and whisker plots.

## Results of Case Study Application to New Zealand

### Categories and Classes

**Fig. 5:**
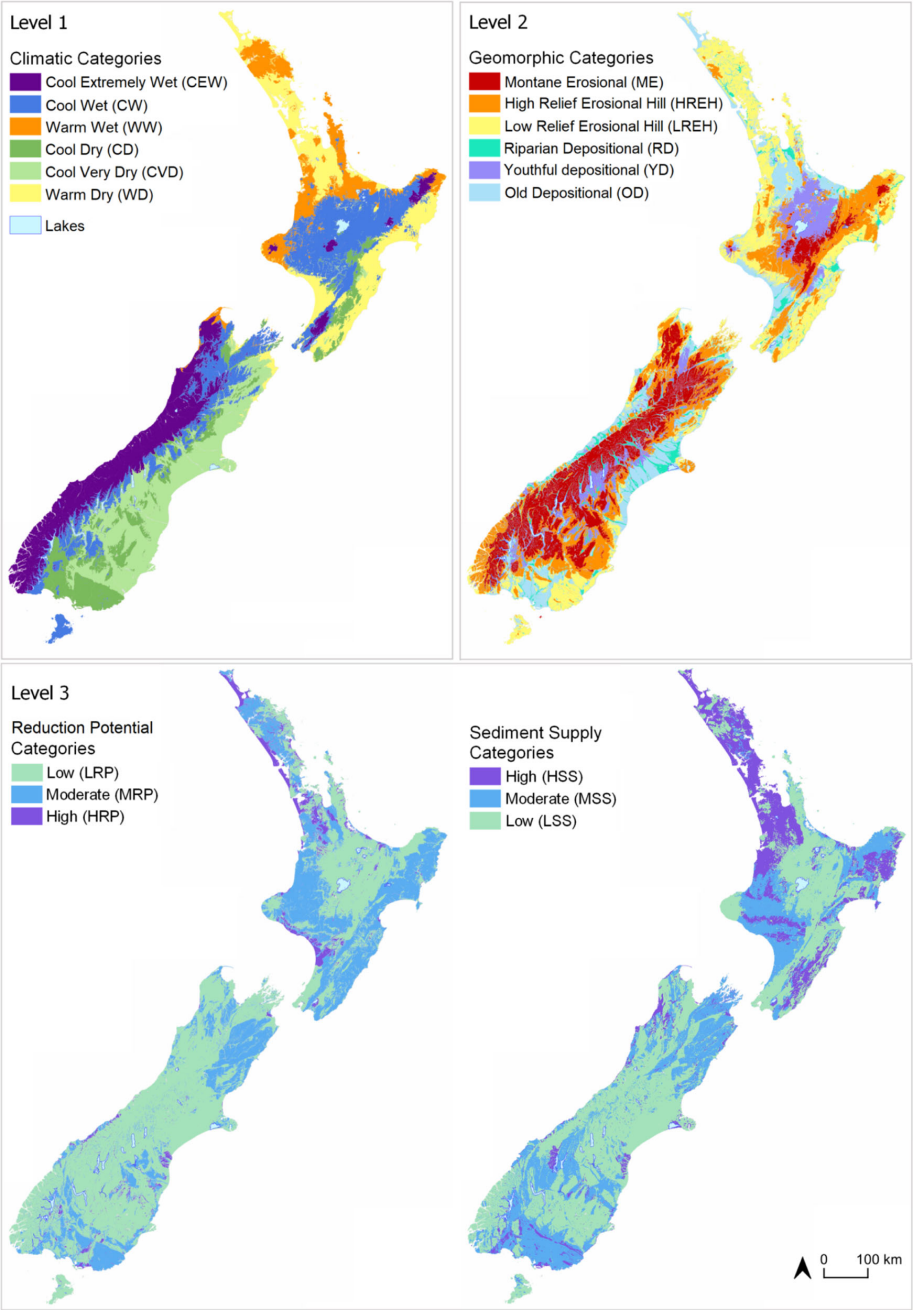
Mapped PEC categories at Level 1 (top left), Level 2 (top right), and Level 3 (bottom). Each category is ordered according to the expected maturity of the waters produced (least (top) to most mature (bottom) in the graphical keys)

**Fig. 6:**
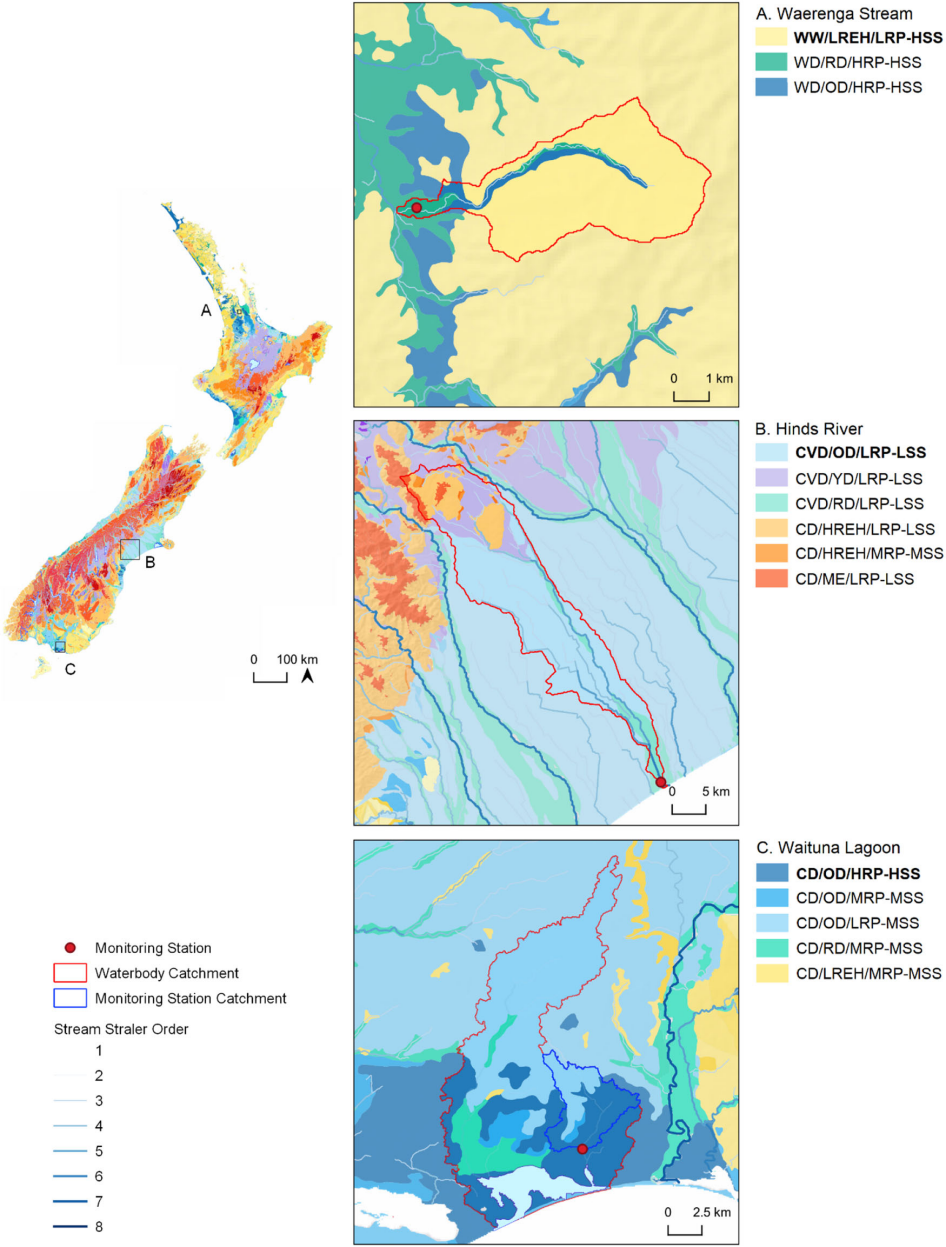
Level 1–3 PEC classes (concatenation of Level 1, 2, and 3 categories) nationally (left) and for three selected smaller domains (right). **A** Waerenga Stream catchment and monitoring station. **B** Hinds River catchment and monitoring station. **C** Waituna Lagoon Catchment, Carran Creek monitoring station and catchment. The dominant class by area is shown in bold in the key. Minor classes (<2%) are not shown in the legend

Maps of PEC categories at Levels 1, 2, and 3 are presented in Fig. 5. Concatenation of Level 1 (Climate) with Level 2 (Geomorphology) categories generates 36 classes at Level 1–2, and concatenation of Level 1–2 classes with Level 3 (Lithology) categories generates 320 PEC classes at Level 1–3 (Fig. 6).

### Results of Statistical Tests

#### Variance partitioning

For the VP analysis, the mean occupancy by the class that each site was assigned to across all three levels of PEC ranged between 93% and 88% (SI 3). Across the water quality variables, the mean numbers of sites were 237, 107, and 115 for PEC levels 1, 1–2, and 1–3, respectively, and the mean number of classes was 6, 10, and 16, respectively. All testable components of variation explained by the VP analysis were significant (*p* < 0.05).

**Fig. 7:**
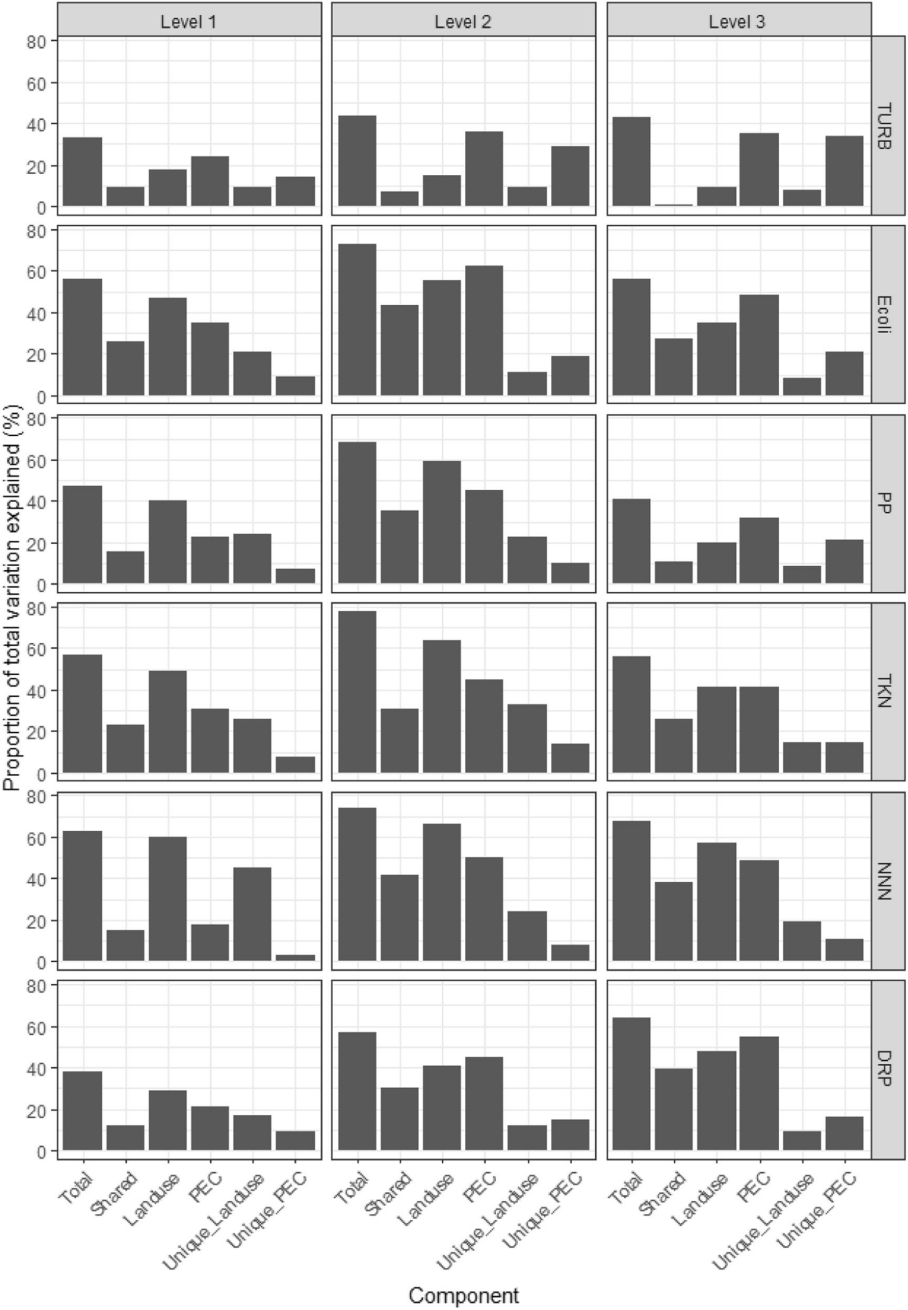
The variance partitioning analysis results show the six components of explained variation (i.e., *R*^2^) by the two sets of explanatory variables for the six water quality variables

The total explained variation (33–78%) and the variation uniquely explained by PEC (3–34%) in the water quality variables increased with each successive level for all variables (Fig. 7 and SI 3). The unique variation explained by land use (8–45%) decreased with each successive increase in the classification hierarchy (i.e., from L1 to L2 and L2 to L3), whereas the unique variation due to PEC increased. Using the ratio of the unique variation associated with PEC to that of land use, PEC explained 0.6 times (×) the variability in NNN and 1.0× (i.e., the same quantum of variability as land use) for TKN. The variation explained by PEC, relative to land use, was 1.8× greater for DRP, 2.3× greater for PP, 2.6× for *E. coli*, and 4.3× for TURB, with a mean value of 2.1× greater across all water quality variables tested.

#### Analysis of variance

**Fig. 8:**
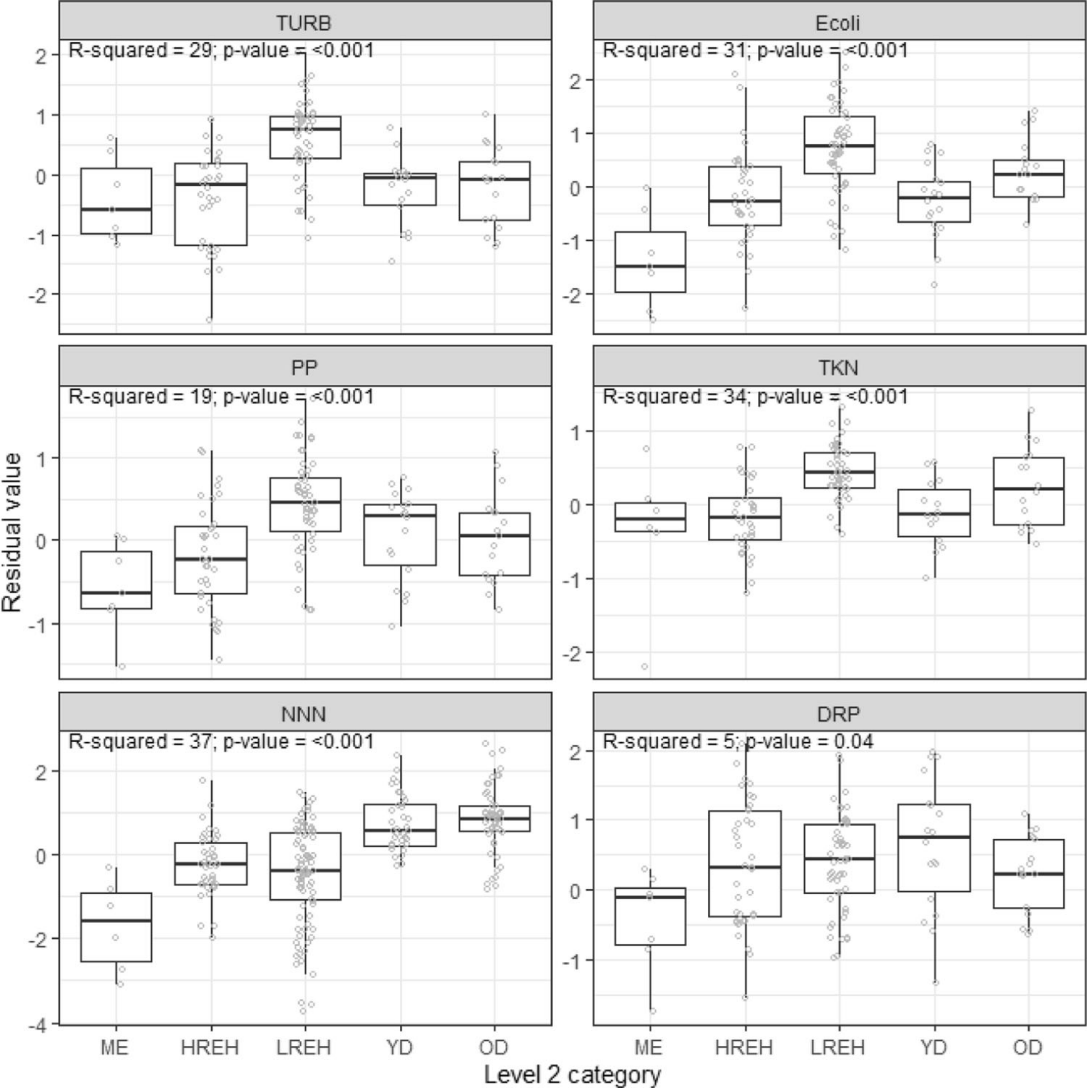
Distributions of residual values of regressions of monitoring station values of each water quality variable against land use grouped by PEC categories at Level 2. The *R*^2^ and *p* values pertain to ANOVAs performed on the data. The black horizontal line in each box indicates the median of site values, and the box indicates the inter-quartile range (IQR). Whiskers extend from the box to the largest (or smallest) values no more than 1.5*IQR from the box. The gray circles indicate the actual data. The categories are ordered from least to most mature waters from left to right

There were significant effects (*p* < 0.05) of PEC categories on all water quality variables at PEC Level 2 (Fig. 8). The variation in the within-category mean values was consistent with the expected susceptibilities outlined in “Application to New Zealand”. For example, the tertiary contaminant nitrate, as indicated by NNN, increased across the geomorphic hydrochemical maturity sequence (i.e., ME to OD). As expected, secondary and tertiary forms of P, as indicated by DRP, also increased across the erosional geomorphic hydrochemical sequence (ME < HREH < LREH), indicating an increasing susceptibility to loss as contact times increase.

Variation in DRP was also consistent with expectations in terms of the contribution of geogenic-P sources across the depositional geomorphic sequence (i.e., geogenic-P YD > > OD; Fig. 8). Low DRP concentrations (second lowest across all geomorphic categories) for the OD category were consistent with expectations of the longest contact times, greatest P-retention, and lowest geogenic-P of all geomorphic categories. In contrast, high DRP concentrations for the YD category were consistent with expectations of a greater geogenic-P source.

As *E. coli* and PP indicated, primary contaminants exhibited trends consistent with the expected susceptibilities outlined in “Application to New Zealand”. Concentrations increased across the erosional geomorphic sequence (ME < HREH < LREH), which is consistent with an increasing abundance of fine textured regolith (Fig. 8). Although turbidity is not purely indicative of primary particulates or inorganic sediments, its increase across the erosional geomorphic sequence (ME < HREH < LREH) is consistent with the expected increase in susceptibility to primary contaminant loss. The loss of secondary forms of N, as indicated by TKN, was also consistent with expectations, increasing across the erosional geomorphic hydrochemical maturity sequence. The lower mean concentration of NNN for the LREH relative to the HREH category is thought to reflect the important role of microbially mediated redox (Level 3), a prominent process for a significant proportion of this category, i.e., denitrification.

**Fig. 9:**
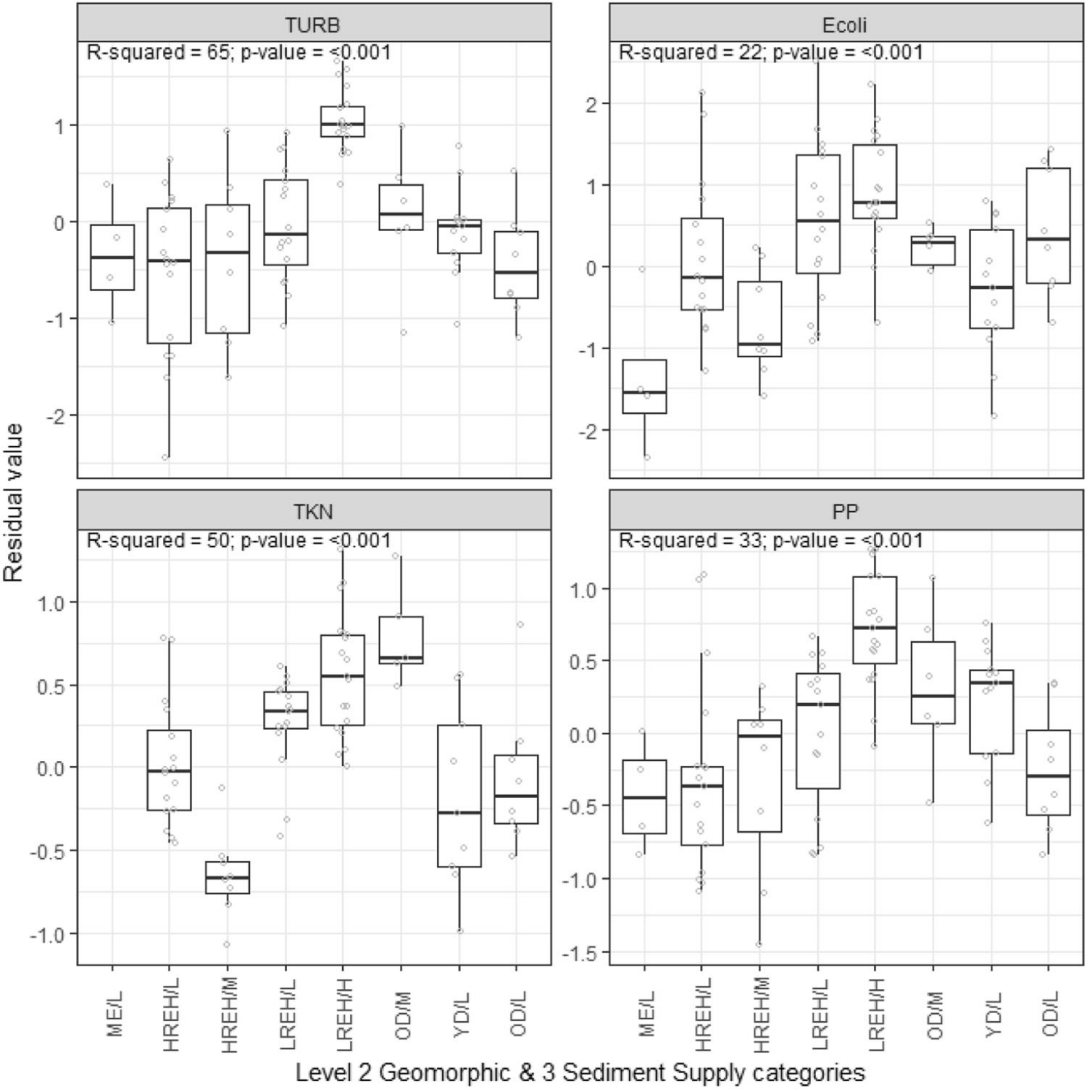
Distributions of residual values of regressions of monitoring station values of each water quality variable against land use grouped by PEC classes generated by the concatenation of Level 2 and 3 categories. The *R*^2^ and *p* values pertain to ANOVAs performed on the data. The black horizontal line in each box indicates the median of site values, and the box indicates the inter-quartile range (IQR). Whiskers extend from the box to the largest (or smallest) values no more than 1.5*IQR from the box. The gray circles indicate the actual data. The categories are ordered from least to most mature waters from left to right

The combination of Level 2 with Level 3 SS categories had significant effects on the water quality variables (Fig. 9). Variation in the within-class mean values was generally consistent with the expected susceptibilities defined in “Level 2: Geomorphic categories” and “Level 3 Lithology”. For example, Fig. 9 indicates increasing and decreasing susceptibility to primary and secondary contaminant loss, as indicated by TURB, *E. coli*, TKN, and PP across the geomorphic hydrochemical maturity sequence. Patterns of susceptibility within each geomorphic category (Level 2) were also consistent with expectations for primary and secondary contaminant loss associated with the Level 3 SS categories (i.e., OD/M > OD/L and LREH/H > LREH/L). For example, greater susceptibility to primary and secondary contaminant loss, as indicated by TURB, *E. coli*, and TKN, for OD/M relative to OD/L and LREH/H relative to LREH/L was consistent with the expectations outlined in “Level 2: Geomorphic categories” and “Level 3 Lithology”. Low susceptibility to agriculturally derived loss of PP and yet elevated PP concentrations for the YD/L class was consistent with the expectations of elevated geogenic-P. However, we note several deviations, with the mean TKN of the HREH/M and that of the OD/M deviating significantly from the expected susceptibilities outlined in “Application to New Zealand”.

**Fig. 10:**
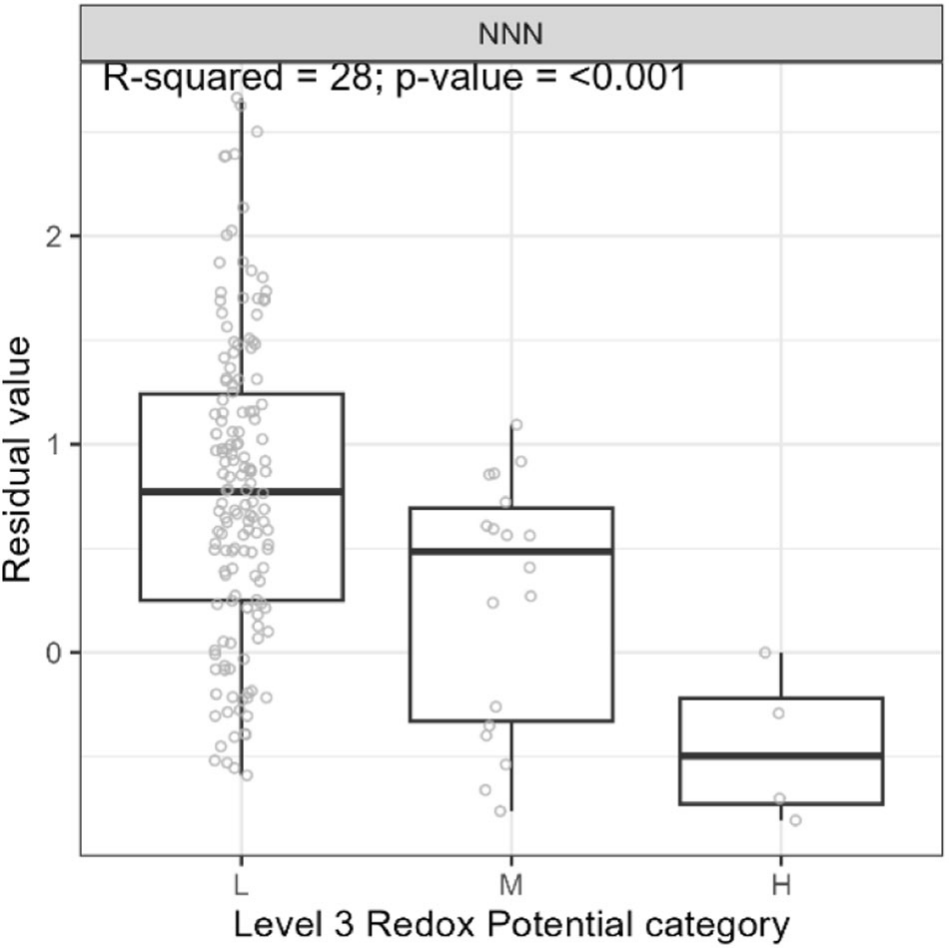
Distributions of residual values of regressions of monitoring station values of NNN against land use grouped by PEC categories at Level 3. The *R*^2^ and *p* values pertain to ANOVAs performed on the data. The black horizontal line in each box indicates the median of site values, and the box indicates the inter-quartile range (IQR). Whiskers extend from the box to the largest (or smallest) values no more than 1.5*IQR from the box. The gray circles indicate the actual data. The categories are ordered from least to most mature waters from left to right

Nitrate concentrations, as indicated by NNN, decreased along the PEC Level 3 RP hydrochemical maturity sequence (L < M < H) (Fig. 10), which was consistent with the expectations set out in ”Application to New Zealand”. Due to the small number of monitoring stations associated with peat wetlands, lignite, or coal-measure-dominated catchments, it was not possible to test the effect of PEC Level 3 RP categories on the concentration of DRP.

## Discussion

PEC builds on the recognition that variation in the landscape-scale controlling factors of climate, geomorphology, and lithology is the cause of variation in hydrochemical maturity (Maher 2010; Sterte et al. 2021; Burt et al. 2022) and susceptibility of different contaminant forms to loss at different scales (Lintern et al. 2018; Liu et al. 2021; O’Sullivan et al. 2023). For example, Becker et al. (2014) identified that natural landscape factors within the 41,000 km^2^ Brazos River watershed, Texas, explained approximately twice the variability in riverine nutrient concentrations as land use.

Our statistical assessment of the proportion of variability explained by the PEC indicates an important component of variability in the type and severity of contaminant loss is due to landscape factors. The ratio of variation that was uniquely attributable to PEC compared to land use varied appreciably by water quality variable. The higher the ratio, the stronger the influence of landscape on agricultural contaminant loss. PEC explained 0.6× (NNN) to 4.3× (TURB) of the variability in surface water quality contaminants relative to land use on its own. The greater contribution of land use to variation in surface water NNN concentrations for surface waters is consistent with the tight coupling between land use intensity and NNN losses. In contrast, our analyses indicate that variation in the loss of P, *E.coli*, and TURB is associated with greater control by landscape factors than by land use, as also noted by others (e.g., Liu et al. 2021). Furthermore, our ANOVA tests and box and whisker plots of water quality variables grouped by categories and classes, and ordered by hydrochemical maturity, demonstrate a strong correspondence with the PEC conceptualization of susceptibilities to contaminant loss.

The PEC builds on efforts to characterize the landscape according to the most important factors known to control water quality (Lintern et al. 2018; Liu et al. 2021; O’Sullivan et al. 2023). However, unlike other landscape classification approaches for water quality, the PEC utilizes the concept of hydrochemical maturity to guide the hierarchal organization, classification, and ranking of factor categories (i.e., according to process magnitude) to produce classes that can be used to explain, at a process level, why variability in contaminant type (primary, secondary, and tertiary) and severity of water quality issues occur. Because PEC elucidates the underlying causes of contaminant loss susceptibility, it can be used to inform targeted and appropriate land management across multiple scales. The PEC supports a global call for a “geosystems” approach to sustainable land management (Izakovičová et al. 2019) by placing land management decisions and tools (including farm-scale nutrient leaching models) within the context of the higher-order environmental factors that control the type and severity of water quality contamination (see also Burkitt and Bretherton 2022).

While monitoring networks are indispensable for conducting state and trend analyses, their broad scale often restricts their capability to elucidate the causes of spatial variation in water quality. Due to their focus on larger catchments, these networks often capture a composite of inputs from diverse landscape units (i.e., discharge from multiple different PEC classes; Fig. 6), complicating the task of discriminating relative contributions, which includes identifying the landscape factors that control the production and loading of different contaminant forms. In situations where the interplay between land use and landscape factors is not adequately understood or spatially resolved, there is a risk that approaches to land management actions will be generalized or approximated. There is a risk that land use is attributed as the sole or dominant driver of spatial variation in the type and severity of water quality issues. This can lead to perverse consequences, including regulations and mitigation actions (including farm system changes), that are inappropriate and, therefore, ineffective (see also O’Sullivan et al. 2023).

The PEC approach addresses these risks by providing land managers with insights into the landscape’s susceptibility to contaminant loss and a model that elucidates the probable causes of this variation across multiple scales. A useful addition to applying PEC to any spatial domain would be the description of the characteristics of classes and the underlying conceptual basis for the processes that produce their specific water quality characteristics. This resource would be useful to land managers and owners who are potential users of PEC and would provide them with an awareness of the most salient landscape factors and processes that, combined with land use, govern agricultural contaminant loss susceptibility.

The PEC classification is limited by the scale and accuracy of the spatial coverages of landscape characteristics it uses. Where spatial coverages are inaccurate or their scale too coarse to capture small catchment or farm scale variability in controlling factors, the classification is unlikely to provide meaningful context to land managers. In places where there is a discrepancy between the expected and observed water quality, hydrochemical and water quality data (e.g., shallow monitoring bores or small catchment and farm scale monitoring of creeks/springs), in conjunction with the physical ground-truthing of controlling factors (e.g., exploration of local soil, or geology), can be utilized to refine the accuracy of the spatial coverages used to inform PEC. Higher-resolution representations of climate, topography (e.g., LiDAR), geology, or soils can be used to improve the resolution and spatial accuracy of the PEC. The insights derived from the interrogation of local anomalies can also be used to refine the conceptual model of the causes of spatial variation in hydrochemical maturity and, consequently, the susceptibility of landscape units to contaminant loss. Applying PEC to other regions globally requires consideration of the climatic, geomorphic, and lithological range, not limited to the consideration of the region’s climatic history, tectonism, and magmatism.

Due to the climatic, geomorphic, and lithological diversity of New Zealand and a bias towards the water quality monitoring of large catchments, our tests were limited in their ability to isolate the effect of individual PEC classes. In particular, patches defined by PEC Level 3 categories were small compared to the resolution of water quality variability indicated by the monitoring network, which limited testing at this level. However, given that the PEC evaluations were consistent with the underlying conceptual model, we are confident that PEC classes are a sound basis for assessing the susceptibility of contaminant loss from different landscapes. More precise testing of PEC would necessitate finer-scale monitoring datasets (e.g., Rissmann et al. 2019).

## Conclusion

The PEC codifies knowledge of the factors controlling the susceptibility of landscape units to the loss of multiple contaminants, enabling patterns to be delineated and the causative processes to be elucidated. Because PEC provides a multi-scale stratification of the landscape, it can provide appropriate and specific information to land and water quality management activities at various spatial scales. PEC also supports a global call for a “geosystems” approach to sustainable land management by placing land management decisions, root zone leaching loss models, tools, and plans within the context of the most important factors that control environmental outcomes.

### Supplementary Information

Supplementary Information
