# Supplementary material for: Physiographic Environment Classification: a Controlling Factor Classification of Landscape Susceptibility to Waterborne Contaminant Loss
Source: Environ Manage. 2024 Mar 5;74(2):230–55. doi: 10.1007/s00267-024-01950-0 (PMC11227452; doi:10.1007/s00267-024-01950-0)
Supplement: Supplementary file 1 — Supplementary Information [file 267_2024_1950_MOESM1_ESM.docx]

# plementary Information

# SI 1. Geomorphic characteristics, hydrochemical maturity, and contaminant susceptibility


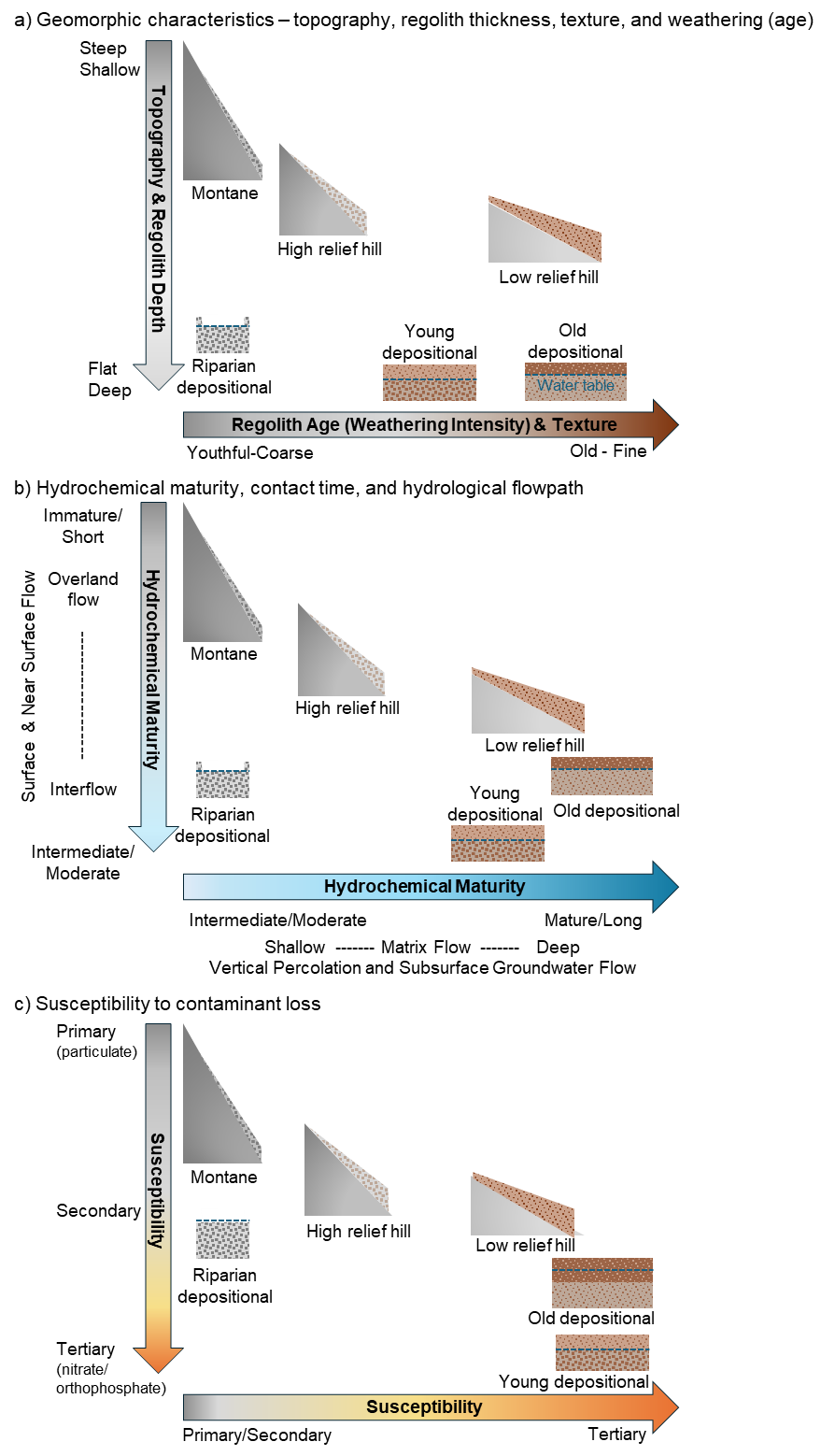


*Figure 11. Schematic of Level 2 geomorphic categories a. Geomorphic characteristics; b. Hydrochemical maturity, and; c. Susceptibility to contaminant loss.*

# SI 2. Map of Monitoring Stations and Upstream Catchment Areas

The dataset was reduced from 885 to 810 sites (Fig. 12).


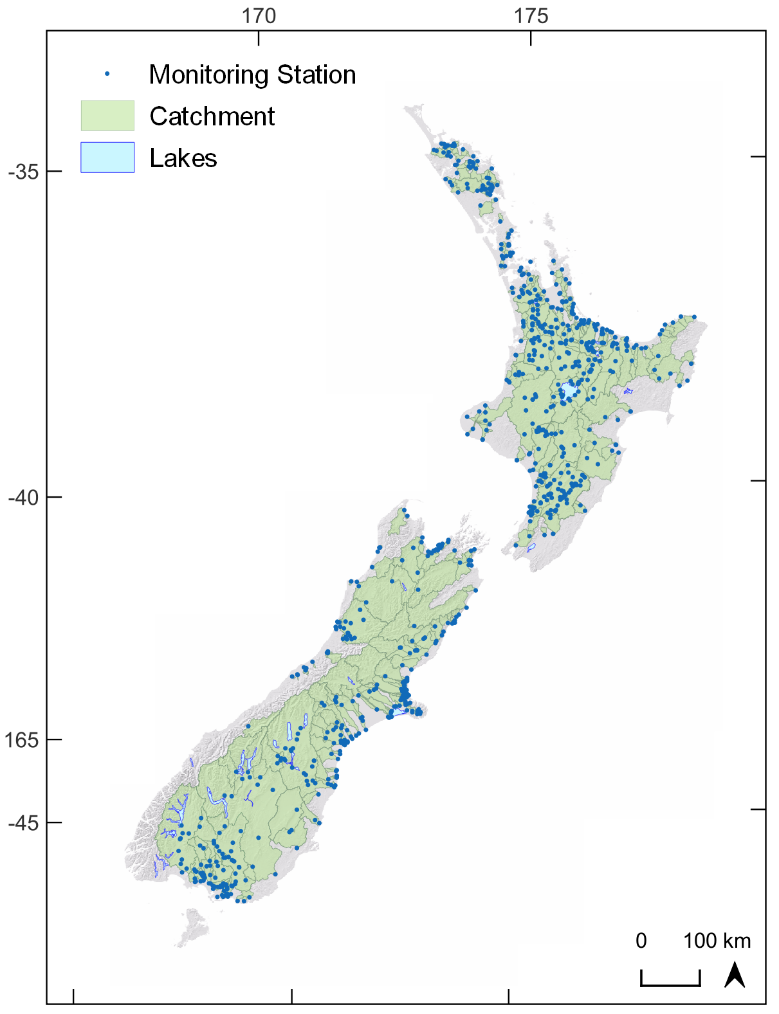


*Figure 12. Water quality indicator sites and upstream catchment areas.*

# SI 3. Tabulated summaries of variance partitioning statistical results

*Table 3. Variance partitioning statistical results.*

| **Name** | Level | No. of Classes | No. of Sites | Mean Occupancy | Total Variance Explained (%) | Land Use Variance Explained (%) | PEC Variance Explained (%) | Shared Variance Explained  (%) | Unique Land Use (%) | Unique PEC  (%) | Unique PEC: Unique LU Ratio |
| --- | --- | --- | --- | --- | --- | --- | --- | --- | --- | --- | --- |
| Turbidity | Level 1 | 6 | 221 | 93 | 33 | 18 | 24 | 9 | 9 | 14 | 1.56 |
| Turbidity | Level 2 | 10 | 96 | 88 | 44 | 15 | 36 | 7 | 9 | 29 | 3.22 |
| Turbidity | Level 3 | 15 | 106 | 92 | 43 | 9 | 35 | 1 | 8 | 34 | 4.25 |
| *E.coli* | Level 1 | 6 | 224 | 93 | 56 | 47 | 35 | 26 | 21 | 9 | 0.43 |
| *E.coli* | Level 2 | 10 | 95 | 88 | 73 | 55 | 62 | 43 | 11 | 19 | 1.73 |
| *E.coli* | Level 3 | 15 | 107 | 91 | 56 | 35 | 48 | 27 | 8 | 21 | 2.63 |
| PP | Level 1 | 6 | 222 | 93 | 47 | 40 | 23 | 16 | 24 | 7 | 0.29 |
| PP | Level 2 | 9 | 91 | 88 | 68 | 59 | 45 | 35 | 23 | 10 | 0.43 |
| PP | Level 3 | 14 | 103 | 92 | 41 | 20 | 32 | 11 | 9 | 21 | 2.33 |
| TKN | Level 1 | 6 | 216 | 93 | 57 | 49 | 31 | 23 | 26 | 8 | 0.31 |
| TKN | Level 2 | 9 | 91 | 88 | 78 | 64 | 45 | 31 | 33 | 14 | 0.42 |
| TKN | Level 3 | 15 | 100 | 92 | 56 | 41 | 41 | 26 | 15 | 15 | 1.00 |
| NNN | Level 1 | 6 | 315 | 94 | 63 | 60 | 18 | 15 | 45 | 3 | 0.07 |
| NNN | Level 2 | 12 | 177 | 90 | 74 | 66 | 50 | 42 | 24 | 8 | 0.33 |
| NNN | Level 3 | 19 | 169 | 92 | 68 | 57 | 49 | 38 | 19 | 11 | 0.58 |
| DRP | Level 1 | 6 | 226 | 93 | 38 | 29 | 21 | 12 | 17 | 9 | 0.53 |
| DRP | Level 2 | 10 | 97 | 88 | 57 | 41 | 45 | 30 | 12 | 15 | 1.25 |
| DRP | Level 3 | 15 | 109 | 92 | 64 | 48 | 55 | 39 | 9 | 16 | 1.78 |
